# Biomineralized outer membrane vesicles for synergistic immuno-photodynamic therapy of oral squamous cell carcinoma

**DOI:** 10.1016/j.ijpx.2026.100537

**Published:** 2026-04-07

**Authors:** Jingyuan Wang, Guanxiong Zhu, Hongru Zhang, Liting Zeng, Da Li, Xinyi Li, Yang Yu, Lu Liang, Lingmin Zhang, Lina Yu

**Affiliations:** a Department of Preventive Dentistry, School and Hospital of Stomatology, Guangdong Engineering Research Center of Oral Restoration and Reconstruction, Guangzhou Medical University, Guangzhou, Guangdong 510182, PR China; b Guangzhou Municipal and Guangdong Provincial Key Laboratory of Molecular Target & Clinical Pharmacology, the State & NMPA Key Laboratory of Respiratory Disease, School of Pharmaceutical Sciences, Guangzhou Medical University, Guangzhou 511436, Guangdong, PR China; c Department of Sports and Health, Guangzhou Sport University, Guangzhou 510500, Guangdong, PR China; d Guangzhou Key Laboratory of Basic and Applied Research of Oral Regenerative Medicine, Guangzhou Medical University, Guangzhou, Guangdong, 510182, PR China,

**Keywords:** Oral squamous cell carcinoma, Photodynamic therapy, Bacterial outer membrane vesicles, Cancer immunotherapy

## Abstract

Immunotherapy against oral squamous cell carcinoma (OSCC) currently exhibits a clinical response rate significantly lower than anticipated, underscoring the need for more effective therapeutic strategies. Outer membrane vesicles (OMVs) represent promising natural immune modulators; however, their systemic application is limited by risks such as cytokine storms and inadequate tumor accumulation. To overcome these challenges, this study developed biomineralized OMVs coated with calcium phosphate (CaP) and subsequently loaded with the photosensitizer chlorin e6 (Ce6) on the surface, termed OMVs@CaP-Ce6 (OCC). The resulting OCC demonstrates enhanced tumor accumulation and responsiveness to acidic conditions, including the tumor microenvironment (TME). Under acidic pH conditions, the CaP shell disintegrates, releasing both OMVs and Ce6 to enable synergistic immunotherapy and photodynamic therapy (PDT). PDT promotes cancer cell apoptosis, while OMVs activate antitumor immunity by modulating immune responses. Studies demonstrate that OCC, as a highly biocompatible and synergistic platform, effectively suppresses tumor growth under laser irradiation by eliminating cancer cells and reversing the immunosuppressive TME to enhance antitumor immunity. This spatiotemporal and biomineralized OCC system maximizes the immunotherapeutic potential of OMVs, overcomes the limitations of conventional monotherapies, and offers a promising alternative strategy for the effective treatment of OSCC.

## Graphical abstract

We have developed Ce6-loaded biomineralized outer membrane vesicles (OCC) specifically engineered to improve the therapeutic efficacy against oral cancer. OCC enables drug release triggered by the tumor microenvironment, effectively minimizing systemic toxicity. Additionally, it combines photodynamic therapy with immune activation, resulting in a synergistic treatment strategy for oral squamous cell carcinoma.

**Figure:**
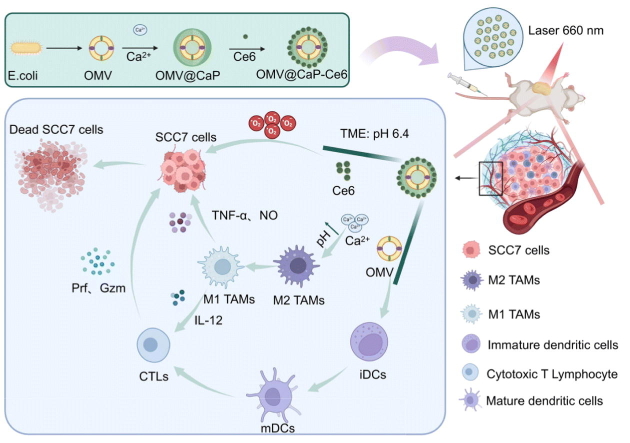

## 1 Introduction

Oral cancer is a common malignant tumor of the head and neck, accounting for approximately 4% of the global incidence of all malignant tumors, with oral squamous cell carcinoma (OSCC) being the predominant pathological subtype (Siegel et al., 2025; Tan et al., 2023). At present, clinical treatments for oral cancer are still dominated by surgery, radiotherapy, and chemotherapy, but all of these modalities have significant limitations. For example, the surgical treatment is prone to impairing patients' oral physiological functions, and the risk of recurrence remains high in advanced cases, radiotherapy and chemotherapy tend to cause damage to normal tissues and are confronted with severe drug resistance challenges (Bai et al., 2024; Xu et al., 2025; Ding et al., 2025a), which makes it difficult to meet the dual clinical demands for therapeutic efficacy and patients' quality of life.

In recent years, the immunotherapy has achieved landmark breakthroughs in the clinical setting due to its unique mechanism of activating the body's innate immune system to achieve specific tumor inhibition (Wu et al., 2023; Lin et al., 2023; Zhang et al., 2023; Postow et al., 2015; Ribas and Wolchok, 2018; Yi et al., 2023). Ranging from immune checkpoint inhibitors (ICIs) that remodel T cell function to chimeric antigen receptor T-cell (CAR-T) therapy that exerts targeted cytotoxicity against hematological malignancies (June et al., 2018); these therapeutic approaches have opened up entirely new paradigms for cancer treatment. However; the inherent immunosuppressive tumor microenvironment (TME) of solid tumors has become a core bottleneck restricting their therapeutic efficacy (Yasuda and Wang, 2024; Mao et al., 2026). As a typical solid tumor of the head and neck, oral cancer has a more heterogeneous TME, characterized by the enrichment of immunosuppressive cells (such as M2-type tumor-associated macrophages) (Zheng et al., 2023) and the accumulation of metabolic byproducts (including lactic acid and kynurenine). These factors collectively form a vicious cycle that hinders immune cell infiltration, suppresses immune cell activation, and induces immune cell functional exhaustion, resulting in a response rate to immunotherapy for oral cancer that is far lower than expected. Thus, there is an urgent need to develop more powerful immune regulation strategies.

Bacterial outer membrane vesicles (OMVs), as natural vesicles spontaneously shed from the outer membrane of Gram-negative bacteria, have emerged as promising carriers in the field of cancer immunotherapy by virtue of their dual properties of “immune regulation and natural delivery” (Toyofuku et al., 2019; Feng et al., 2022; Sun et al., 2024; Tang et al., 2023; Pei et al., 2024). On the one hand, OMVs inherently carry pathogen-associated molecular patterns (PAMPs), such as lipopolysaccharides (LPS) and lipoproteins, which can efficiently activate dendritic cells (DCs) via pattern recognition receptors (e.g. TLR4). As initiators of adaptive immunity, activated DCs significantly enhance antigen presentation efficiency, thereby promoting the proliferation of effector T cells and their infiltration into tumor tissues. On the other hand, the nanoscale size (20–200 nm) and membrane structural features of OMVs endow them with favorable biocompatibility and cell-penetrating ability, laying a natural foundation for drug loading and targeted delivery (Zheng et al., 2024; Li et al., 2025). Nevertheless, the clinical translation of OMVs still faces two core challenges: first, during systemic administration, exposed PAMPs are prone to triggering systemic nonspecific inflammatory responses, and may even induce cytokine storms, which severely limits their safe dosage (Qing et al., 2020; Kim et al., 2017; Ban et al., 2023). Second, the natural OMVs are easily cleared rapidly by the mononuclear phagocyte system (MPS), leading to insufficient accumulation at tumor sites and thus hindering their precise immune regulatory effects.

The rise of biomineralization technology provides a crucial strategy for addressing the aforementioned challenges in the clinical translation of OMVs. By coating OMVs with a pH-sensitive inorganic shell, multiple functional optimizations can be achieved simultaneously. The physical barrier formed by this inorganic shell can effectively shield the nonspecific immune activation induced by PAMPs, thereby significantly reducing the systemic toxicity of OMVs. Meanwhile, the acidic conditions unique to the tumor microenvironment (pH 6.0–6.5) can trigger the degradation of the shell, enabling the responsive release of OMVs and substantially improving their delivery efficiency at tumor sites. This TME-responsive property also lays a foundation for the precise regulation of local tumor immunity.

Although mineralized OMVs have demonstrated potential for tumor immune regulation, the single immunotherapy remains challenged to achieve efficient radical treatment of oral cancer. Notably, most oral cancers are superficial tumors, which makes them ideal candidates for photodynamic therapy (PDT). PDT kills tumor cells by generating reactive oxygen species (ROS) via photosensitizers under light irradiation at specific wavelengths (Ding et al., 2025b; Ding and Kim, 2026), offering high targeting ability and minimal damage to normal tissues (Ding et al., 2025c; Ding et al., 2026). A number of studies have verified its value in the treatment of oral cancer. For instance, some research has applied hematoporphyrin derivatives as photosensitizers in PDT for in situ oral cancer lesions, achieving precise elimination of primary tumor cells without causing obvious damage to the oral mucosa (Yu et al., 2024). For residual micro-lesions after oral cancer surgery; Ce6-mediated PDT has also exhibited favorable clearance effects; as the ROS it generates can directly kill residual tumor cells and simultaneously release tumor-associated antigens; providing a prerequisite for subsequent immune activation (Xu et al., 2024; Li et al., 2024a; Xu et al., 2021; Ding et al., 2022; Nie et al., 2020; Wang et al., 2024a). However, traditional Ce6-mediated PDT also has limitations, including insufficient light penetration in tumor tissues, low photosensitizer accumulation efficiency, and the suppression of PDT-induced immune responses by the immunosuppressive TME, all of which limit its therapeutic efficacy (Wang et al., 2024b; Xu et al., 2017; Li et al., 2024b).

Based on the above research background and the disease characteristics of oral cancer, this study proposes a synergistic therapeutic strategy integrating engineered mineralized outer membrane vesicles with Ce6-mediated PDT. With OMVs as the core, this strategy achieves shielding of systemic toxicity and tumor microenvironment-responsive release of OMVs via biomineralization modification with a pH-sensitive calcium phosphate (CaP) shell, and simultaneously loads Ce6 to construct the OMVs@CaP-Ce6 composite nanosystem. By leveraging the TME responsiveness, the mineralized shell can be degraded in the TME, releasing OMV and Ce6 at the tumor site. This system further synergistically enhances photodynamic killing and OMV-mediated immune activation, providing a novel insight into the efficient treatment of OSCC.

## 2 Materials and methods

### 2.1 Materials

Chlorin e6 (Ce6) was purchased from MedChem Express (China); DMEM/F-12 medium, fetal bovine serum (FBS), phosphate-buffered saline (PBS), and penicillin/streptomycin were all purchased from Gibco (USA); LB broth was purchased from Guangdong Huankai Microbial Science and Technology Co., Ltd.

### 2.2 Cell culture

**Scheme 1:**
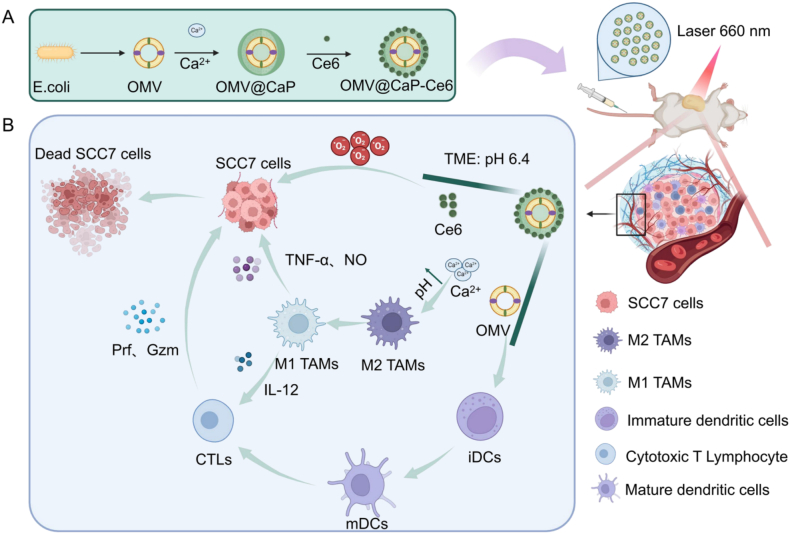
The scheme diagram for the preparation of OCC and the effect on tumor inhibition. (A) The preparation of OMV. (B) The application and biological effects of OMV in vivo.

The mouse squamous cell carcinoma cell line SCC7 (Squamous Cell Carcinoma 7) was purchased from the American Type Culture Collection (ATCC). SCC7 cells were routinely cultured in complete DMEM/F-12 medium (Gibco) supplemented with 10% fetal bovine serum (FBS, Gibco) and 1% Penicillin-Streptomycin (Gibco), and maintained in a cell incubator (Thermo Scientific) at 37 °C with 5% CO₂ and saturated humidity (Scheme 1).

### 2.3 Bacterial culture

The bacterial strain used for outer membrane vesicle (OMV) isolation in this study was *Escherichia coli* BL21, which was purchased from China Center of Industrial Culture Collection (CICC). Single colonies were randomly picked from the agar plate of the Escherichia coli strain, inoculated into the LB liquid medium, and cultured with shaking at 37 °C and 200 rpm for 12 h. Subsequently, the bacterial culture was transferred to fresh LB medium at a 1:100 inoculation ratio, and the culture was shaken until the cells reached the logarithmic growth phase (OD₆₀₀ ≈ 1.0).

### 2.4 Detailed procedures for OMVs preparation

Four liters of bacterial culture were collected, and gradient centrifugation was performed sequentially (300 ×*g*, 1000 ×g, 3000 ×g, and 10,000 ×g for 20 min each at 4 °C) to remove bacterial cells. The supernatant was filtered through a 0.22 μm pore-size vacuum filter membrane, followed by concentration using an ultrafiltration centrifugal tube with a 50 kDa molecular weight cut-off (Millipore). Following initial OMV concentration using a 50 kDa ultrafiltration membrane, the retentate undergoes two to three iterative diafiltration cycles with phosphate-buffered saline (PBS). In each cycle, the concentrated sample is diluted with PBS and then re-concentrated via centrifugation—a process that effectively dilutes and removes soluble contaminants, including monomeric and small-aggregate forms of LPS. The concentrated sample was centrifuged at 100,000 ×*g* for 3 h at 4 °C using a Beckman Type 90 Ti fixed-angle rotor (k-factor = 25) with 12.5 mL tubes in a Beckman Ultracentrifuge Optima XPN-100 Model 60,028 to pellet OMVs. The OMV pellet was resuspended in PBS and filtered through a 0.22 μm filter membrane to remove residual bacteria or debris. All operations were conducted under sterile conditions to prevent contamination. The protein concentration of OMV samples was determined using an enhanced BCA Protein Quantification Kit (Beyotime), the yield of the obtained vesicles was approximately 4.2 mg/L. After aliquoting, the samples were stored at −80 °C for subsequent use.

### 2.5 Preparation of OMV@CaP-Ce6

First, OMVs containing 1 mg of total protein were dispersed in 1 mL of PBS (Gibco) and incubated overnight in a constant-temperature shaking incubator (200 rpm) at 37 °C to reach equilibrium. Subsequently, 20 μL of CaCl₂ solution (1 mol/L) was added to the reaction system, and the reaction was carried out with continuous magnetic stirring (500 rpm) at 37 °C for 2 h to promote the biomineralization of CaP on the surface of OMVs. After the reaction was completed, the mixture was washed three times with PBS via differential centrifugation (12,000 ×*g*, 10 min, 4 °C) to remove unbound calcium and phosphate ions, yielding purified OMV@CaP composites (Feng et al., 2022; Chen et al., 2024). The protein concentration of OC samples was determined using an enhanced BCA Protein Quantification Kit (Beyotime), approximately 170–220 μg of protein was encapsulated within the calcium phosphate shell.

Subsequently, the prepared OMV@CaP composites were mixed with chlorin e6 (Ce6) at a mass ratio of 1:6, and the reaction was conducted with magnetic stirring (500 rpm) at 37 °C for 2 h under light-protected conditions. The reaction product was also washed three times with PBS (12,000 ×g, 10 min, 4 °C), and finally resuspended in PBS. The resulting nanocomposites were stored at 4 °C in a light-protected environment for subsequent use. All operations were performed under sterile conditions in a biosafety cabinet to ensure no microbial contamination during the experiment.

### 2.6 Characterization of OMV@CaP-Ce6

The zeta potential, hydrodynamic particle size, and polydispersity index (PDI) of OMV, OMV@CaP (OC), and OMV@CaP-Ce6 (OCC) nanoparticles were determined using dynamic light scattering (DLS, Malvern, UK). To simulate the serum-containing physiological environment in vivo, long-term DLS measurements were performed to monitor the particle size and dispersibility of nanoparticles, so as to evaluate their colloidal stability under physiological conditions. For this purpose, OCC samples were dispersed in prepared DMEM and kept under dark conditions. Particle size and PDI were then recorded at 0, 12, 24, 36, 48, 60, and 72 h. All measurements were performed at 25 °C, with three parallel replicates for each sample.

A 10 μL aliquot of the suspension containing OMV, OC, or OCC nanoparticles was dropped onto a 200-mesh carbon-supported copper grid and allowed to stand at room temperature for 10 min to ensure full adsorption of the nanoparticles. Subsequently, the grid was negatively stained with a 2% (*w*/*v*) phosphotungstic acid solution (pH 7.0) for 2 min, followed by gentle rinsing with ultrapure water three times (5 min each time) to remove excess stain. After blotting the edge liquid with filter paper, the sample was placed in a desiccator and left to stand overnight at room temperature. Once completely dried, the morphological characteristics of the samples were observed and imaged using a field-emission transmission electron microscope (JEM-1400Flash, JEOL, Tokyo, Japan) at an acceleration voltage of 80 kV.

Samples of free Ce6, pure OMVs, OC, and OCC were prepared separately and adjusted to appropriate concentrations in PBS. The UV–Vis spectrophotometer was set to a scanning range of 200–800 nm, with PBS as the blank control. The absorption spectra of each sample were measured sequentially.

Equal amounts (20 μg) of protein samples from OMVs, OC, and OCC were mixed with 5 × SDS-PAGE loading buffer (containing 2% SDS and 5% β-mercaptoethanol) at a ratio of 4:1. The samples were heated in a metal bath at 99 °C for 10 min to achieve complete protein denaturation. The denatured samples were loaded onto a 12% separating gel for SDS-PAGE and electrophoresed at 120 V. After electrophoresis, the gel was stained with Coomassie Brilliant Blue R-250 staining solution (0.1% *w*/*v*) with shaking at room temperature for 1 h. The stained gel was then destained with destaining solution until the background became transparent and protein bands were clearly visible. The gel was placed on a white background plate and imaged with a smartphone at a fixed distance (30 cm) under constant illumination. To reduce glare, the flash was turned off, and the smartphone lens was kept perpendicular to the gel surface during imaging.

Encapsulation efficiency (EE) and Drug loading content (DLC) optimization was performed using different mass ratios (Ce6/OC = 1:1–10:1). After centrifugation at 12,000 ×*g* for 15 min at 4 °C, the content of free Ce6 in the supernatant was determined via UV–Vis spectrophotometry at 402 nm, and the loading efficiency was calculated using the following formula,

$$\mathrm{EE}\;\left(\%\right)=\left(\text{Loaded Ce6 amount}/\text{Total Ce6 initially added}\right)\times 100\%$$

$$\mathrm{DLC}\;\left(\%\right)=\left(\text{Loaded Ce6 amount}\right)/\left(\text{Total nanoparticle weight}\right)\times 100\%$$

All experiments were performed in triplicate (results expressed as mean ± standard deviation), and samples were washed with PBS to remove surface-adsorbed drug before measurement.

OCC composites were dissolved in PBS and then loaded into a dialysis bag with a 3.5 kDa molecular weight cut-off. The dialysis bag was immersed in 40 mL of release medium (maintained at 37 °C) with pH 6.4 (to simulate the tumor microenvironment) or pH 7.4 (to simulate physiological conditions), and the drug release experiment was conducted in a constant-temperature shaker at 220 rpm. At present time points (0–96 h), 1 mL of the release medium was sampled (with an equal volume of fresh medium added simultaneously to maintain a constant volume). Calcium ion concentration was first determined using an atomic absorption spectrometer (AAS) at the characteristic wavelength of 422.7 nm for calcium, where samples were collected following the same protocol. Subsequently, the cumulative release amount of Ce6 was quantified using a microplate reader (BioTek) at 402 nm, with samples collected using the same collection method. To investigate the release mechanism of Ce6, the in vitro release data were fitted to three mathematical models: zero-order (Q_t_ = k_0_t), Higuchi (Q_t_ = k_H_t^1/2^), and Korsmeyer-Peppas (Q_t_ = kt^n^). The model with the highest correlation coefficient (R^2^) was considered the best fitting model. For the Korsmeyer-Peppas model, only data points with cumulative release ≤60% were used to calculate the release exponent nn, as per the model's applicability criteria. All fitting was performed by linear regression analysis.

A standard hemolysis assay was performed using fresh anticoagulated whole blood from BALB/c mice to systematically evaluate the blood compatibility of OMV, OC, and OCC nanomaterials at concentrations of 0.1–1.0 mg/mL. The brief procedure was as follows: 20 μL of whole blood was co-incubated with 980 μL of the test sample at 37 °C for 3 h. After centrifugation, the absorbance of the supernatant was measured at 540 nm, with PBS and deionized water serving as negative and positive controls, respectively.

### 2.7 Quantitative analysis of cell viability

To explore the effect of an acidic microenvironment (pH 6.4) on SCC7 cell survival, which would facilitate subsequent in vitro experiments simulating the acidic tumor environment, we first conducted relevant assessments. Subsequently, the cytotoxic effects of formulations at different concentrations on SCC7 cells were systematically evaluated using the CCK-8 assay (all groups were incubated for 8 h, with two conditions investigated: laser irradiation and non-irradiation). Additionally, to assess the biocompatibility of OMVs, we determined their cytotoxicity at different concentrations on dendritic cells (DCs). Cell viability in all these experiments was quantified by measuring absorbance at 450 nm using a multi-functional microplate reader (BioTek, USA).

### 2.8 Cellular binding in vitro

We systematically assessed the binding behavior of OCC nanoparticles to SCC7 cells using confocal laser scanning microscope (CLSM, Zeiss LSM900) and flow cytometry (CytoFLEX S). Specifically, SCC7 cells were seeded at a density of 2 × 10^5^ cells/well in confocal dishes. After a 24 h incubation period, the cells were exposed to a medium containing nanoparticles with Ce6 equivalent concentrations ranging from 1 to 2.5 μg/mL, and cultured for durations ranging from 1 to 8 h separately at pH 7.4 and pH 6.4. Following fixation with 4% paraformaldehyde, the cells were stained with Actin-Tracker Green and DAPI to evaluate subcellular localization. The binding efficiency was then quantified via flow cytometry.

### 2.9 In vitro cytotoxicity evaluation

SCC7 cells were seeded in confocal dishes and 6-well plates at a density of 2.5 × 10^5^ cells/well, followed by pre-incubation for 24 h under pH 7.4 and pH 6.4 conditions to ensure full adherence. Subsequently, the cells were treated with different formulations, including PBS (negative control), OMVs, Ce6, OC, OCC, and OCC pre-treated at pH 7.4, for 8 h. After treatment, the cells were irradiated with a 660 nm laser (2 W/cm^2^) for 3 min. Then, the cells were incubated with Calcein-AM/PI double-staining reagent (Beyotime, China) at 37 °C for 30 min under light-protected conditions. Finally, cell living/death was assessed using a confocal laser scanning microscope (CLSM).

For flow cytometry analysis, SCC7 cells in 6-well plates were digested with EDTA-free trypsin for 30 s, and the digestion was terminated by adding complete medium containing serum. The cell suspension was collected by gentle pipetting and centrifuged at 2000 rpm for 5 min at room temperature. After discarding the supernatant, the cells were washed once with pre-cooled PBS and resuspended in 50 μL of Binding Buffer. Subsequently, 5 μL of Annexin V-FITC and 5 μL of propidium iodide (PI) staining solution were added, respectively. After gentle mixing, the cells were incubated at room temperature for 20 min under light-protected conditions. Immediately after completing all staining steps, the cells were analyzed by flow cytometry (CytoFLEX S).

### 2.10 Experimental design for reactive oxygen species (ROS) generation detection

To systematically evaluate the intracellular ROS level in SCC7 cells under different treatment conditions, cells were first seeded in confocal dishes at a density of 2 × 10^5^ cells/dish and cultured for 24 h under pH 7.4 and pH 6.4 conditions to achieve full adherence. The cells were then divided into 12 experimental groups for treatment: PBS (negative control), PBS + Laser, OMVs, OMVs + Laser, Ce6, Ce6 + Laser, OC, OC + Laser, OCC, OCC + Laser, OCC (under pH 7.4 conditions), and OCC + Laser (under pH 7.4 conditions). Each group was treated for 4 h, and all operations were performed under light-protected conditions to eliminate interference from ambient light with the experimental results.

After treatment, the cells were incubated with DCFH-DA fluorescent probe (10 μM) at 37 °C for 30 min under light-protected conditions to specifically label intracellular ROS. For groups requiring light treatment, a 660 nm laser (2 W/cm^2^) was used for irradiation for 3 min to activate the photosensitizer. Immediately thereafter, detection was performed using a CLSM. By quantitatively analyzing the fluorescence intensity of each group, the difference in ROS generation efficiency induced by different treatment combinations with or without laser irradiation could be accurately evaluated.

### 2.11 Evaluation of dendritic cell (DCs) maturation

DCs cultured for 7 days were collected after trypsin digestion and counted. Then, the DCs were seeded into 48-well plates (3 × 10^5^ cells/well). The experimental groups were treated with complete medium containing 100 μg/mL OMVs, OC, or OCC, while a 1 μg/mL LPS-stimulated group was set as the positive control. All groups were co-incubated for 24 h at 37 °C with 5% CO₂. After incubation, DCs were collected and centrifuged at 2300 rpm for 7 min to obtain cell pellets.

The collected DC pellets were resuspended, and fluorescently labeled antibodies against mouse Cluster of differentiation 11c (CD11c), cluster of differentiation 80 (CD80), and major histocompatibility complex class II molecule (MHC-II) were added, respectively. The cells were incubated at 4 °C for 30 min under light-protected conditions for surface labeling. After antibody incubation, the cells were washed twice with pre-cooled PBS to remove unbound antibodies. Finally, the cells were resuspended in 300 μL of PBS, filtered through a 40 μm cell strainer, and transferred to flow tubes. A flow cytometer was used to detect the expression levels of co-stimulatory molecule (CD80) and major histocompatibility complex class II molecule (MHC-II) on the surface of DCs in each group, so as to evaluate the regulatory effect of different nanotherapeutics on the maturation status of DCs. The experiment was performed in three independent replicates, and at least 10,000 CD11c^+^ cells were collected for statistical analysis in each detection.

### 2.12 Animal experiments

Female BALB/c mice aged 5–6 weeks and weighing 18–20 g were used in this study. All mice were housed in a specific pathogen-free (SPF) environment. All animal experiments were approved by the Animal Ethics Committee of Lai'an Technology (Guangzhou) Co., Ltd. (Approval No. G2025040) and strictly conducted in accordance with the Guidelines for Ethical Review of Laboratory Animal Welfare (GB/T 35892–2018).

### 2.13 Dynamic monitoring of in vivo drug distribution via in vivo imaging system

After random grouping, tumor-bearing mice were anesthetized with 2% isoflurane and intravenously injected via the tail vein with different formulations, the Ce6 group and the OCC group (Ce6 equivalent dosage: 2 mg/kg); the control group was injected with an equal volume of PBS. A PerkinElmer IVIS Lumina LT in vivo imaging system (USA) was used to perform whole-body fluorescence imaging at 1, 2, 4, 8, 24, and 48-h post-administration.

To comprehensively evaluate the tissue distribution characteristics of the drug, the mice were euthanized after the final imaging, and major organs (heart, liver, spleen, lung, and kidney) were dissected for ex vivo imaging. All fluorescence images were quantitatively analyzed using Maestro V3.0. A professional analysis software was used to measure the mean fluorescence intensity of the region of interest (ROI).

### 2.14 in vivo antitumor efficacy

A squamous cell carcinoma model was established by subcutaneously inoculating 1 × 10^6^ SCC7 cells into the right axilla of each mouse. When the tumor volume reached approximately 100 mm^3^, the mice were randomly divided into 8 groups (*n* = 5 per group): PBS group (negative control), PBS + Laser group (phototherapy control), Ce6 group (photosensitizer control), Ce6 + Laser group (single photodynamic therapy), OMV group (empty vector control), OC group (OMV@CaP complex), OCC group (OMV@CaP-Ce6 composite formulation), and OCC + Laser group (combined therapy group).

Mice in each group were intravenously injected with the corresponding formulation via the tail vein every 48 h (Ce6 equivalent 2 mg/kg; OMV equivalent 30 μg protein). At 4 h post-injection, the tumors were irradiated with a 660 nm laser (power density: 2 W/cm^2^, duration: 10 min; the laser spot diameter covered the entire tumor surface). A total of 5 treatment cycles were performed. During the treatment period, the tumor length (L) and width (W) were measured regularly, and the tumor volume was calculated using the formula: V = W^2^ × L/2 (Tomayko and Reynolds, 1989). After the experiments were finished, the mice were euthanized. The tumors and major organs (heart, liver, spleen, lung, and kidney) were collected. The samples were fixed in 4% paraformaldehyde, embedded in paraffin, and subjected to hematoxylin-eosin (H&E) staining for histopathological evaluation, Ki-67 immunohistochemistry for cell proliferation, and TUNEL assay for cell apoptosis. Meanwhile, systemic toxicity was evaluated by complete blood count analysis and detecting serum biochemical indicators including alanine transaminase (ALT), aspartate transaminase (AST), blood urea nitrogen (BUN), creatinine (Cr), etc.

### 2.15 Statistical analysis

Data analysis was performed using GraphPad Prism 8.0 software. Data are presented as mean ± SD (*n* = 3, biological replicates), unless otherwise specified. Two-tailed student's *t*-test or one-way analysis of variance (ANOVA) was performed for comparisons between two or more groups, supplemented with multiple comparison correction (Bonferroni correction). A statistically significant difference was considered when *P* < 0.05 (*P < 0.05, **P < 0.01, and ***P < 0.001, unless otherwise specified).

## 3 Results

### 3.1 Preparation and characterization of OCC

We prepared and characterized OCC. First, we successfully extracted OMVs from the culture medium of Gram-negative bacteria. To verify the purity of the obtained OMVs (i.e., the efficacy in removing live bacteria and free LPS), OMV samples were inoculated on LB agar plates, and no colony growth was observed after 24 h. Meanwhile, Coomassie brilliant blue staining was conducted to confirm that there was no significant difference in protein expression among different batches (Fig. S1). Concurrently, the LPS content in the supernatant obtained after ultracentrifugation was determined to be <0.1 EU/mL using a limulus amebocyte lysate (LAL) assay, which meets the safety requirements for both in vitro cellular experiments and in vivo animal studies.

Next, we coated OMVs with a CaP shell and loaded Ce6 via absorption to form the OCC nanosystem. The encapsulation efficiency of Ce6 gradually increased from 1:1 to 1:6 (indicating insufficient Ce6 addition at lower ratios), while no significant improvement was observed when the ratio was increased to 1:10; when the mass ratio of Ce6 to OMV@CaP was 6:1, the loading efficiency reached as high as 86% (Fig. S2), We choose this mass ratio for the following experiments.

**Fig. 1:**
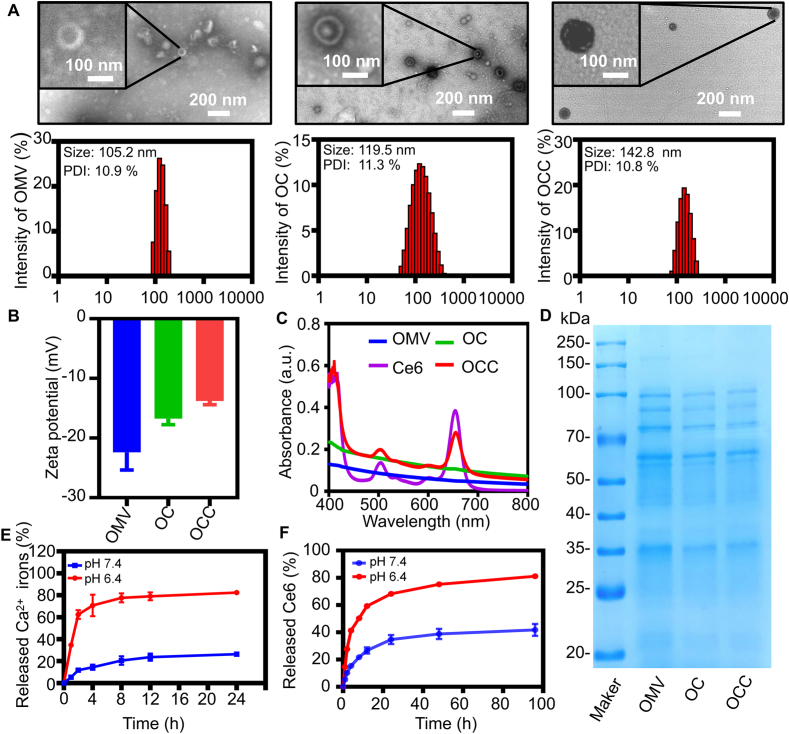
Characterizations of OC and OCC. (A) TEM analysis and the size distribution of OMV, OC and OCC. (B) The zeta potential of OMV, OC and OCC. (C) The UV–Vis Absorption Spectra of OMV, 6CE, OC, and OCC. (D) Coomassie Brilliant Blue Staining of OMV, OC, and OCC. (E) The cumulative release of Ca^2+^ at different pH conditions. (F) The cumulative release of OCC. (For interpretation of the references to colour in this figure legend, the reader is referred to the web version of this article.)

To systematically verify the synthesis efficiency and key structural characteristics of the nanoparticles, we conducted a series of characterizations to analyze their physicochemical properties. Transmission electron microscopy (TEM) and DLS analysis indicated that OMVs showed a spherical structure with an average particle size of 105.2 nm (Fig. 1A). After the coating with CaP shell (OC), the surface of the nanoparticles showed more distinct with the size increasing to 119.5 nm (Fig. 1A). The loading of Ce6 on OC also presented a dark surface, and the size increased to 142.8 nm (Fig. 1A). The zeta potential of OMVs, OC, and OCC was corresponding to −22.4 mV, −16.8 mV, and − 13.8 mV, respectively (Fig. 1B). This increase in particle size and reduction in zeta potential implied the successful coating of the CaP shell and effective modification of Ce6 molecules. We measured the particle size changes of nanoparticles in medium containing 10% fetal bovine serum (FBS) at 0, 24, 48, and 72 h. The results showed no obvious increase in particle size (Size: 143.01 ± 3.93 nm) within 72 h (PDI < 0.20 at all time points) and no significant aggregation, indicating excellent colloidal stability in serum-containing medium (Fig. S3). To confirm that the successful loading of Ce6, UV–Vis spectroscopy was performed and indicated that OCC maintained the characteristic absorption peak of Ce6 between 450 and 700 nm and showed a minor redshift (Fig. 1C), implying that the successful loading of Ce6. SDS-PAGE analysis (Fig. 1D) indicated that the most of proteins-derived from OMVs were retained in OCC, which demonstrated that the process of mineralization with CaP and loading of Ce6 did not affect the natural components of OMVs. Thus, the biological function of OMVs as an immune-activating carrier was preserved.

We also investigated the pH-responsive release profiles of the nanomedicine under pH 7.4 (normal physiological condition) and pH 6.4 (acidic tumor microenvironment). The formulation exhibited obvious pH-dependent release behavior. At pH 7.4, the nanocarrier was stable with slow Ce6 release (34.70% at 24 h). In comparison, the CaP shell rapidly degraded under weakly acidic condition (pH 6.0), thus weakening coordination adsorption and accelerating drug release to 68.20% within 24 h. Meanwhile, Ca^2+^ release from OCC was significantly higher at pH 6.4 than at pH 7.4, which favored enhanced antitumor efficacy in the acidic tumor microenvironment (Fig. 1E and F). These results confirmed the successful preparation of OCC with desirable acidic responsiveness.

The in vitro release data at pH 7.4 and 6.4 were fitted to zero-order, Higuchi, and Korsmeyer-Peppas models, with the Korsmeyer-Peppas model providing the best fit, Korsmeyer-Peppas fitting yielded release exponent (n) values of 0.629 and 0.540 at pH 7.4 and pH 6.4, respectively (Table S1). Both values indicate anomalous (non-Fickian) transport, with the lower n value at pH 6.4 reflecting a greater contribution of Fickian diffusion. This pH-dependent shift is consistent with the acid-sensitive dissolution of the calcium phosphate shell in the acidic tumor microenvironment, which accelerates drug release and supports targeted therapeutic applications.

### 3.2 In vitro biocompatibility evaluation of OCC

A hemolysis assay was conducted to assess the hemocompatibility of OCC (Fig. S4). The positive control (Triton-X) caused complete red blood cell (RBC) lysis, whereas OCC-treated groups at different concentrations for 3 h showed results comparable to the negative control, characterized by a clear supernatant and intact RBC pellet. Quantitative analysis (Fig. S4B) revealed hemolysis rates of 5.1 ± 0.3% at 100 μg/mL and 2.5 ± 0.2% at a lower concentration after 3 h. According to biomaterial criteria, where a hemolysis rate below 5% indicates good hemocompatibility, OCC exhibits minimal hemolytic activity. These results suggest that OCC has excellent hemocompatibility and favorable in vitro biocompatibility, supporting its safety for biomedical applications.

To clarify the baseline viability of oral cancer cells in a simulated acidic tumor microenvironment (pH 6.4) and physiological pH (7.4), a systematic evaluation was conducted using the Cell Counting Kit-8 (CCK-8) assay. Additionally, the effect of the OCC nanotherapeutic combined with photodynamic therapy (PDT) on cell viability was assessed. The results showed that short-term exposure (48 h) to an acidic environment did not significantly reduce the baseline viability of SCC7 cells (Fig. S5A), thereby eliminating the interference of pH itself on subsequent drug toxicity evaluation.

In the absence of laser irradiation, no significant changes in cell viability were observed when the concentration of OCC ranged from 0.2 to 3.2 μg/mL. However, after laser irradiation, cell viability decreased in a dosage-dependent manner with increasing drug concentration, and the half-maximal inhibitory concentration (IC₅₀) decreased to 0.52 μg/mL. These findings indicate that PDT can significantly enhance the tumor-killing activity of the nanotherapeutic (Fig. S5B).

Furthermore, to comprehensively evaluate the in vitro biocompatibility of OCC, the cytotoxicity of OCC against dendritic cells (DCs) was further detected at the cellular level using the CCK-8 assay. The viability of DCs in all concentration groups remained above 85%, and there was no statistically significant difference compared with the blank control group (0 μg/mL) (supplement with statistical analysis if available, e.g., *P* > 0.05) (Fig. S5C). These findings indicate that OCC has no obvious cytotoxicity toward immune cells (DCs).

### 3.3 Cell binding to OSCC

Cellular binding serves as a crucial parameter for assessing the prospective therapeutic efficacy of nanoparticles. At pH 6.4, SCC7 cells were exposed to OCC (equivalent Ce6: 1.0–2.5 μg/mL) for 8 h. Confocal Laser Scanning Microscopy (CLSM) revealed weak red fluorescence predominantly near the cell membrane at 1.0 μg/mL Ce6. As the concentration increased, fluorescence intensity significantly enhanced and showed a more widespread distribution throughout the cytoplasm at 2.5 μg/mL. Flow Cytometry (FACS) analysis confirmed a concentration-dependent binding. The binding efficiency reached 92.5% with no signs of saturation at 2.5 μg/mL Ce6; this concentration of OCC was therefore chosen for subsequent evaluations. Compared to OCC at pH 7.4, the pH 6.4 condition exhibited significantly higher and more uniformly distributed cytoplasmic fluorescence, as verified by CLSM and FACS, confirming that the acidic microenvironment (pH 6.4) facilitated OCC binding.

**Fig. 2:**
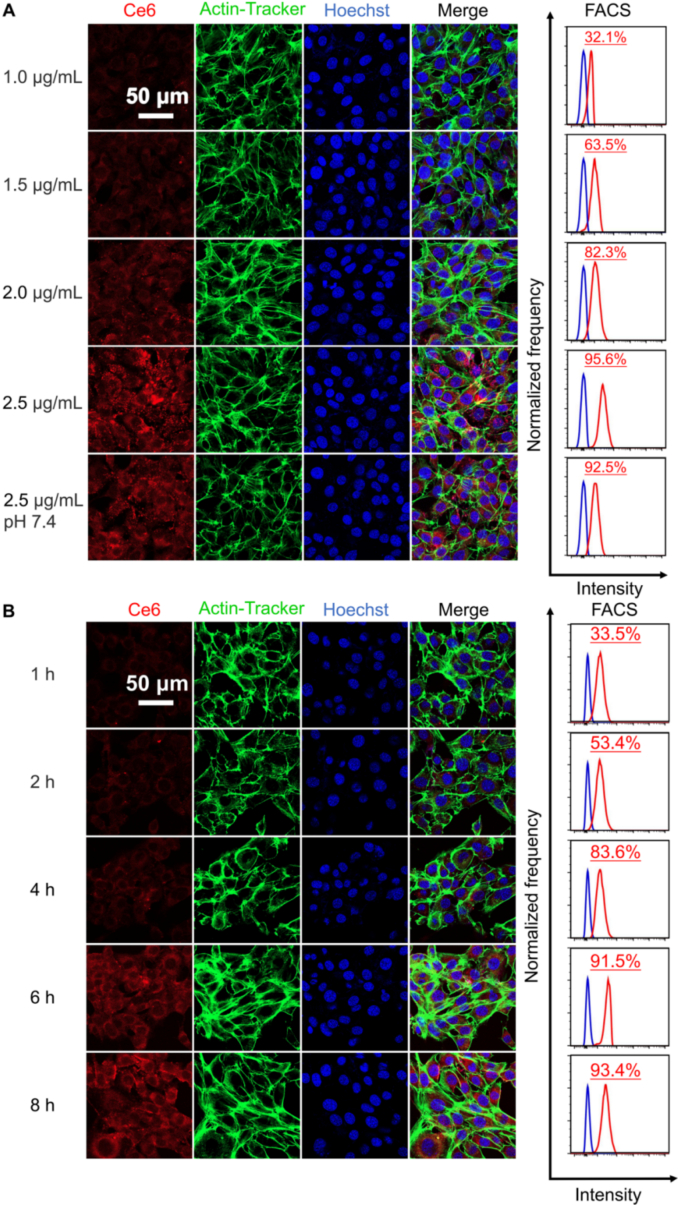
In vitro assessment. Conditions for cellular binding under various conditions by CLSM and flow cytometry. (A) SCC7 cells were incubated to OCC at equivalent Ce6 concentrations of 1.0–2.5 μg/mL for 8 h at pH 6.4; an additional group treated with 2.5 μg/mL OCC (equivalent Ce6) was established at pH 7.4 as a control. (B) SCC7 cells were incubated with OCC (2.5 μg/mL Ce6) at pH 6.4 for periods ranging from 1 to 8 h.

SCC7 cells were incubated with OCC (2.5 μg/mL Ce6) at pH 6.4 for periods ranging from 1 to 8 h. CLSM showed weak fluorescence, indicating low binding, at 1–2 h; a marked increase at 4 h; and a plateau at 8 h, with no difference compared to 6 h. FACS analysis demonstrated a typical time-dependent binding pattern. Ultimately, OCC containing 2.5 μg/mL Ce6 following an 8 h incubation was employed for subsequent experiments (Fig. 2).

### 3.4 Biological effects induced by OCC

**Fig. 3:**
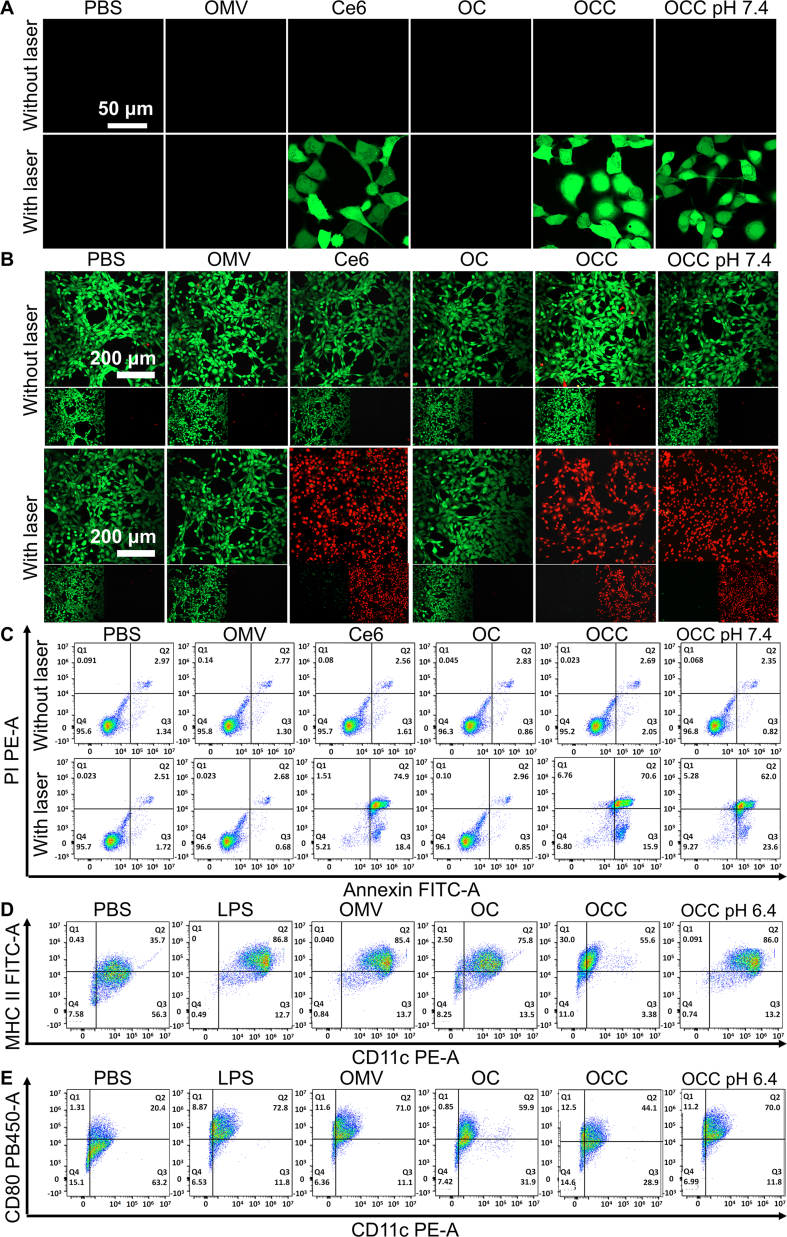
In vitro evaluation of the biological effects induced by OCC. ROS level detection, Live/Dead staining, apoptosis, and in vitro activation of immature dendritic cells. SCC7 cells were treated with PBS, OMV, Ce6, OC, OCC for 8 h, with an additional OCC group at pH 7.4; all groups were set with 660 nm laser irradiation and non-irradiation controls. (A) Cellular ROS level determination by DCFH-DA method. (B) Fluorescence microscopy images of SCC7 cells stained with Calcein-AM/PI. (C) Apoptosis detection of SCC7 cells. (D) In vitro activation of dendritic cells.

Using a DCFH-DA ROS detection kit, we assessed the levels of ROS generated in SCC7 cells following exposure to various experimental treatments. Detection with the DCFH-DA probe revealed that only weak green fluorescence was observed in all groups without laser irradiation (Fig. 3A), indicating that no significant ROS generation was induced by different formulations, and the intracellular redox homeostasis was not affected significantly. After the exposure to laser irradiation, the intracellular green fluorescence intensity in the Ce6, OCC (pH 6.4), and OCC (pH 7.4) groups was significantly enhanced, indicating that laser irradiation can effectively activate Ce6 to generate ROS and further trigger cellular oxidative stress responses. Among them, the laser-irradiated OCC (pH 6.4) group showed much stronger green fluorescence intensity than the one in pH 7.4, suggesting higher intracellular ROS levels. These results indicate that the acidic microenvironment can synergistically promote the release of Ce6 from OCC and its cellular uptake. Upon laser excitation, a large amount of ROS is generated, which induces cell death by disrupting intracellular redox homeostasis and damaging the biomacromolecules, such as DNA and proteins. This is fully consistent with the enhanced killing effects observed in the aforementioned cell viability detection and apoptosis experiments.

Live-dead cell staining was conducted using calcein and propidium iodide (PI), with green fluorescence indicating viable cells and red fluorescence signifying dead cells. Live/Dead staining (Fig. 3B) showed that without laser irradiation, the cells treated with different formulations including PBS, OMV, Ce6, OC, OCC, and OCC (pH 7.4), exhibited predominantly uniform green fluorescence with minimal red fluorescence (only scattered in naturally apoptotic cells) and maintained intact spindle or polygonal morphology. After laser irradiation, obvious red fluorescence was observed in the Ce6 alone, OCC (pH 6.4), and OCC (pH 7.4) groups. Ce6-loaded nanoparticles exhibited low cytotoxicity, thereby minimizing adverse effects on normal cells in the absence of laser irradiation; in contrast, they demonstrated a potent tumor cell-killing effect upon exposure to laser light.

Apoptosis assay results demonstrated that without laser irradiation, there was no significant difference in the total apoptosis rate among all groups (Fig. 3C). However, after laser irradiation, the Ce6 alone, OCC (pH 6.4), and OCC (pH 7.4) groups all showed significant pro-apoptotic effects. Notably, the killing efficiency of the OCC group under simulated acidic conditions was significantly higher than that of the OCC group at pH 7.4. This difference is presumably attributed to the acidic environment promoting the degradation of the OC shell and accelerating the release of Ce6, which provides a preliminary basis for enhancing the efficacy of photodynamic therapy (PDT) through subsequent combination with laser irradiation, and further verifies the regulatory role of the pH responsiveness of the nanocarrier in drug-mediated killing effects. Collectively, these results demonstrate that Ce6-loaded nanoparticles exert a potent cytotoxic effect on SCC7 cells specifically when exposed to laser irradiation. In an in vitro simulation of an acidic environment, the OCC + Laser group showed an enhanced apoptotic response compared to control groups.

The antitumor mechanism of *E. coli*-derived OMVs is their ability to activate the host immune system. This study examined the immunostimulatory effects of OCC in an acidic tumor microenvironment (pH 6.4) on dendritic cell (DC) maturation by analyzing surface markers. Immature DCs were co-incubated with various formulations for 24 h, and flow cytometry revealed that all formulations significantly increased CD80^+^ and MHC-II^+^ DCs compared to controls, with OCC (pH 6.4) showing effects similar to OMVs (Fig. 3D and E). Thus, all formulations effectively mature DCs, enhancing their T cell interaction and antigen presentation. Thus, OMVs and OC induce DC maturation without extra activators, and mineralization does not hinder their immunostimulatory capacity, especially OCC in acidic conditions, which supports future combination with photodynamic therapy.

The in vitro results demonstrate that OCC exerts synergistic antitumor and immunostimulatory effects in a laser- and pH-dependent manner. Without laser irradiation, the nanomaterial shows negligible cytotoxicity against SCC7 cells, thereby avoiding off-target damage. However, under laser activation, it induces dosage-dependent tumor cell apoptosis via an intracellular ROS burst. Notably, this antitumor activity is significantly enhanced in the acidic tumor microenvironment (pH 6.4) due to acid-responsive CaP shell degradation and accelerated Ce6 release. Furthermore, OCC retains the inherent immunostimulatory activity of bacterial OMVs by effectively promoting DC maturation through upregulation of surface costimulatory (CD80^+^) and antigen-presenting (MHC-II^+^) molecules, which lays the foundation for subsequent adaptive immune responses. Importantly, mineralization modification does not impair this immune activity. OCC at pH 6.4 exhibits DC maturation efficiency comparable to native OMVs.

### 3.5 In vivo distribution

**Fig. 4:**
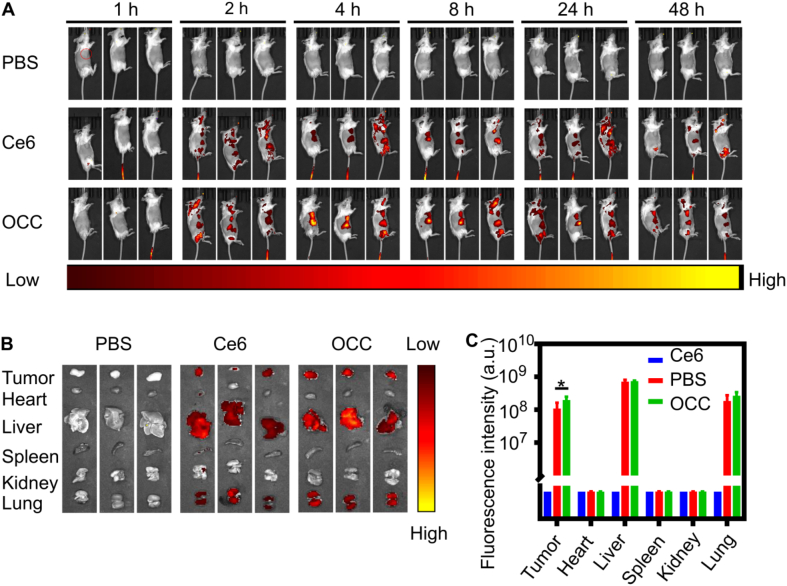
In vivo biodistribution and homotypic targeting evaluation in BALB/c mice (*n* = 3) administered with Ce6 and OCC. BALB/c mice (*n* = 3 per group) were administered with Ce6 or OCC to evaluate in vivo biodistribution and homotypic targeting of the formulations. (A) Analysis of in vivo biodistribution of fluorescent nanoparticles. (B, C) Ex vivo imaging of major organs and corresponding quantitative evaluation. All data are presented as mean ± standard deviation. *P < 0.05 between two groups.

In vivo imaging systems allow real-time monitoring of fluorescent signals in living tissues, aiding drug distribution analysis. This study used the intrinsic fluorescence of Ce6 to observe the distribution of free Ce6 and OCC nanoparticles in BALB/c tumor-bearing mice. After intravenous injection, the fluorescent images were captured at various time points. At 2 h, signals were widespread, mainly in well-perfused tissues, with no tumor accumulation. However, after the injection for 4 h, both groups showed increased accumulation at the tumor site, which might be due to the EPR effect, with OCC showing significantly higher intensity and better retention at 48 h (Fig. 4A). Quantitative analysis indicated that OCC's tumor intensity was 1.8 times greater than free Ce6, while both groups showed signals in the liver and kidneys, aligning with drug metabolism patterns (Fig. 4B-C). In summary, the preferential accumulation and prolonged retention of OCC nanoparticles in tumor tissues provide a solid foundation for achieving effective laser-activated photodynamic therapy against OSCC.

### 3.6 In vivo antitumor efficacy

**Fig. 5:**
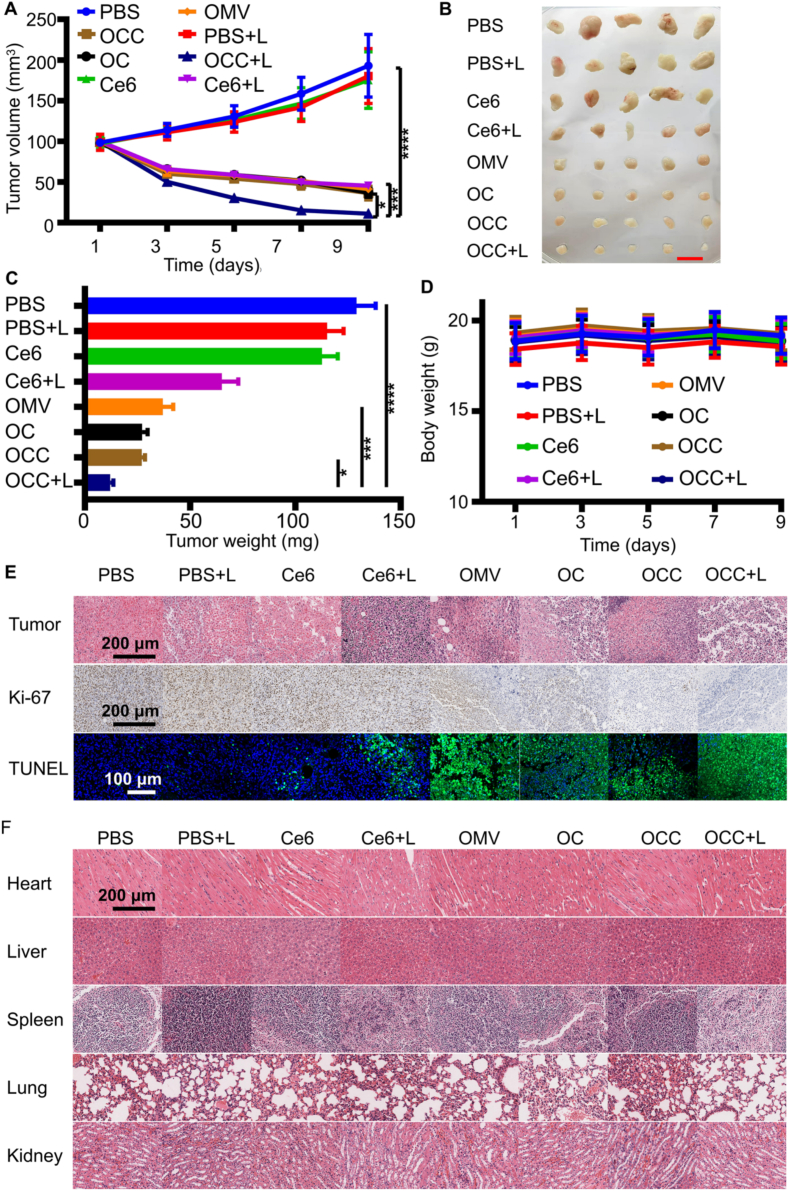
Antitumor efficacy in SCC7 tumor-bearing BALB/c mice (*n* = 5) after tail vein injection of different formulations. SCC7 tumor-bearing BALB/c mice were randomly divided into 8 groups and treated via tail vein injection: PBS group, PBS + Laser group, Ce6 group, Ce6 + Laser group, OMV group, OC group, OCC group, and OCC + Laser group. The administration dosage of Ce6, OC, and OCC was equivalent to Ce6 at 2.5 mg/kg, while the dosage of OMV was 30 μg per mouse. (A) Photographs of excised tumors after different treatments. (B) Dynamic changes in tumor volume. (C) Tumor weights recorded on Day 11 at the end of treatment. (D) Body weight changes of mice in different treatment groups. (E) Representative pathological sections of tumor tissues stained with H&E, Ki-67, and TUNEL. (F) H&*E*-stained sections of major organs. All data are presented as mean ± standard deviation. ns indicates no significant difference; inter-group comparisons: * *P* < 0.05, ** *P* < 0.01, *** *P* < 0.001, and **** *P* < 0.0001.

To investigate the in vivo antitumor efficacy of OMVs@CaP/Ce6 (OCC), a tumor-bearing mouse model was established, and various formulations were administered (Fig. 5A-C). As shown in Fig. 5A-C, the OCC + Laser group achieved the smallest tumor volume, indicating the most effective tumor inhibition. Notably, statistical analysis revealed that the OCC + Laser group exhibited a significant difference (*P* < 0.05) compared with the OCC alone group, and a highly significant difference (*P* < 0.001) compared with the Ce6 + Laser group. Among the formulations containing Ce6, OCC + Laser demonstrated greater antitumor efficacy than free Ce6, confirming its enhanced tumor suppression. These differential results demonstrate that the antitumor effect of OCC + Laser is jointly mediated by tumor immune activation and photodynamic therapy, and the synergistic interaction between these two mechanisms results in maximal antitumor efficacy. Body weight monitoring indicated that all treatments were safe, with no significant differences in body weight changes among tumor-bearing mice across all groups (Fig. 5D).

Histological observations revealed significant necrosis in the OCC + Laser group, while control groups showed mild necrosis. The expression of Ki67 was notably lower in laser-treated groups, especially in the OCC + Laser group (Fig. 5E and Fig. S6A). Although OCC in the absence of laser irradiation also showed some inhibition on Ki67, the one was inferior to the OCC treated group. In comparison, other groups including PBS, PBS + L, free Ce6, Ce6 + L, OMV, and OC showed much lower inhibition of Ki67 compared with OCC treated groups. TUNEL assay indicated few apoptotic cells in the non-laser groups, but the OCC + Laser group possessed the highest apoptosis rate, accounting for approximately 80%, with many cells labeled in green fluorescence (Fig. 5E and Fig. S6B). These data further confirm that OCC exerts effective tumor inhibition in vivo when combined with laser irradiation. This effect may be attributed to its excellent tumor-targeting ability, pH-responsive drug release, and the synergistic effects of immune activation and photodynamic therapy, suggesting the great potential of OCC for oral squamous cell carcinoma therapy.

To assess the biosafety of the formulations, the pathological examinations of tumor tissues and major organs revealed no significant pathological abnormalities or tissue damage between treatment and control groups, indicating that OCC treatment did not induce pathological changes in normal organs (Fig. 5F). Complete blood count (CBC) and serum biochemical analyses indicated the absence of severe systemic toxicity, showing normal blood cell counts (Fig. S7) and markers of liver and kidney function (Fig. S8). Together, these findings suggest that OCC may be a safe and effective therapeutic agent in the tested model.

### 3.7 In vivo immune response

**Fig. 6:**
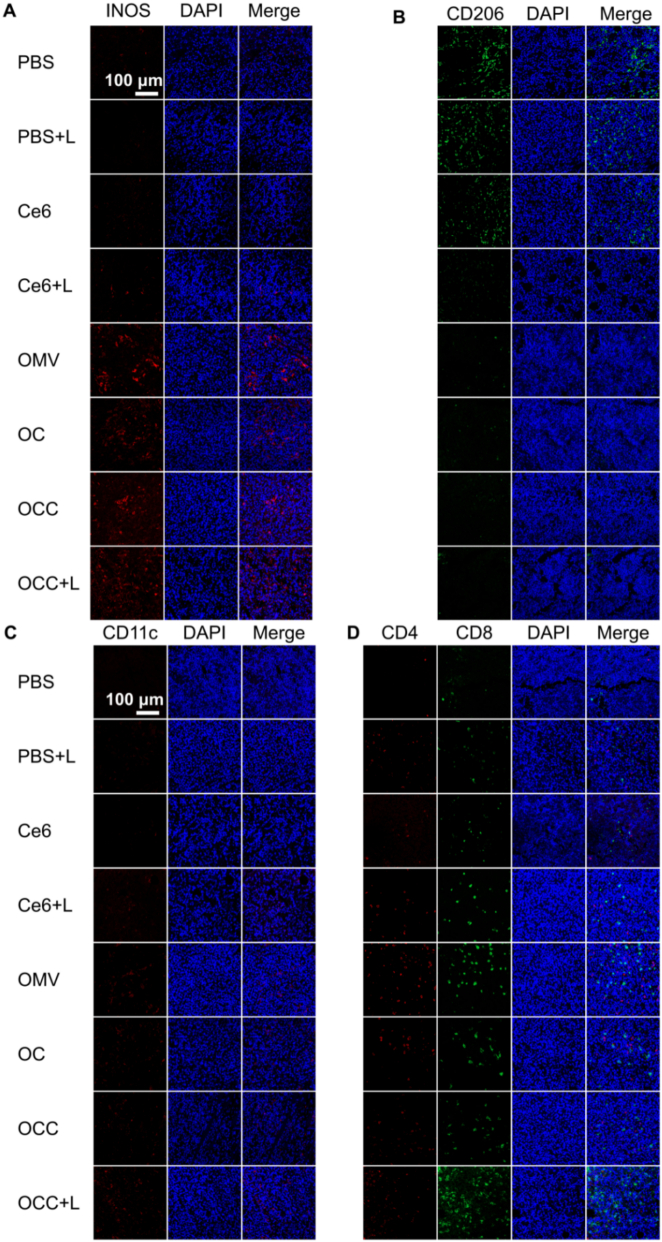
Immunofluorescence analysis of tumor tissues after treatment with OCC-based nanoparticles. (A, B) Macrophage labeling in tumor tissues. (C) Dendritic cell labeling in tumor tissues. (D) T cell labeling in tumor tissues.

To clarify the regulatory effects of different treatments on the tumor immune microenvironment, the immunofluorescence staining was performed on tumor tissue sections from tumor-bearing mice (See Fig. 6). Specific markers were used to identify different immune cell subsets: CD206 for M2-type macrophages, iNOS for M1-type macrophages, CD11c for dendritic cells (DCs), and CD4/CD8 for T cells. Among all immune cell indicators, the OMV group exhibited excellent immune cell enrichment characteristics: weak fluorescent signals for CD206^+^ M2-type macrophages, while strong signals and high cell numbers were observed for iNOS^+^ M1-type macrophages; CD11c^+^ DCs showed prominent positive signals, with wide distribution and high infiltration within tumor tissues; CD4^+^ T cells and CD8^+^ T cells (especially CD8^+^ cytotoxic T cells) displayed strong signals and large quantities, resulting in the highest overall infiltration level.

Notably, the OCC combined with laser treatment group exhibited significantly enhanced immune cell characteristics compared with both the OC and OCC groups. Specifically, the signal of iNOS^+^ M1-type macrophages was markedly restored, accompanied by a notable increase in cell numbers, indicating a robust activation of the pro-inflammatory response. In contrast, the signal of CD206^+^ M2-type macrophages, which are typically associated with anti-inflammatory functions, was attenuated, suggesting a shift away from immunosuppressive activity. Furthermore, the positive signal for CD11c^+^ dendritic cells (DCs) was notably strengthened, characterized by an expanded infiltration range and increased cell counts, highlighting enhanced antigen-presenting capabilities. The signal of CD4^+^ T cells also showed enhancement, but more importantly, the signal of CD8^+^ cytotoxic T cells was substantially elevated, with a significant increase in cell numbers and a denser distribution throughout the tumor microenvironment. The overall immune cell enrichment in this group was superior to that in the OMV group, and the cytotoxicity of CD8^+^ T cells was more pronounced, indicating a more aggressive immune response against the tumor. These observations were further validated by quantitative analysis of immunofluorescence signals in each group, conducted using ImageJ software, as illustrated in Fig. S9. The immunofluorescence staining analysis clearly demonstrated that OMVs alone can effectively regulate the tumor immune microenvironment. However, it was observed that the CaP coating and Ce6 loading slightly weakened this regulatory effect. In contrast, the combination of OCC with laser treatment restored and even enhanced the immune regulatory effect. This synergistic approach works by inducing M1 polarization of macrophages, promoting the infiltration of dendritic cells, and enriching the infiltration of CD8^+^ cytotoxic T cells, ultimately leading to a significant improvement in the tumor immune microenvironment.

## 4 Discussion

The clinical treatment of oral squamous cell carcinoma (OSCC) has long been plagued by multidimensional bottlenecks (Zhuang et al., 2024). Surgical intervention is prone to impairing oral physiological functions and is associated with a persistently high recurrence rate in advanced cases; radiotherapy and chemotherapy are confronted with the challenges of normal tissue toxicity and drug resistance. In addition; the extensive enrichment of M2-type tumor-associated macrophages (TAM-M2) in the immunosuppressive TME severely restricts the response rate of immunotherapy (Liu et al., 2020; Yao et al., 2022; Yang et al., 2020). Meanwhile, although Ce6-mediated photodynamic therapy (PDT) alone is inherently suitable for OSCC due to its superficial anatomical characteristics (Cen et al., 2023); its efficacy is limited by insufficient photosensitizer accumulation; poor light penetration in tumor tissues; and the suppression of immune responses by the immunosuppressive TME (Ma et al., 2023). Thus, achieving simultaneous, precise tumor elimination, and reversal of TME immune suppression has become the key to overcoming the predicament of OSCC treatment. The nanosystem OCC developed in this study integrates the immune-regulatory capacity of biomineralized OMVs with the tumor-killing effect of Ce6-PDT, providing a novel synergistic paradigm for OSCC therapy.

OCC achieves targeted breakthroughs in the traditional therapeutic bottlenecks of OSCC through the precise coupling of structure and function. The biomineralization modification of the pH-sensitive calcium phosphate (CaP) shell addresses two core drawbacks of natural OMVs: on the one hand, the physical barrier formed by the CaP shell shields the non-specific immune activation induced by pathogen-associated molecular patterns (PAMPs) on the surface of OMVs, significantly reducing systemic inflammatory toxicity and overcoming the limitation of restricted safe dosage of natural OMVs; on the other hand, it can undergo responsive degradation in the acidic TME of tumors (pH 6.0–6.5), enabling site-specific release of OMVs and Ce6 at tumor loci. Meanwhile, it reduces OMV clearance by the mononuclear phagocyte system (MPS), thereby markedly enhancing the accumulation of OCC in tumor tissues. From the perspective of therapeutic efficacy, Ce6 generates ROS upon light irradiation to directly and precisely kill OSCC cells, and its minimally invasive property avoids surgical tissue damage, the core mechanism of photodynamic therapy (PDT) relies on the synergistic effects of photosensitizers, specific wavelength light, and oxygen. The accessibility of oxygen directly determines the efficiency of reactive oxygen species (ROS) generation, while the hypoxic microenvironment commonly present in solid tumors has long been a critical bottleneck limiting the efficacy of conventional PDT (Cai et al., 2025); the released OMVs can activate DCs, promote the infiltration of effector T cells, and reprogram the phenotype of TAM-M2 to reverse the immunosuppressive TME, making up for the shortcomings of insufficient immune activation by PDT alone and limited killing ability by the single immunotherapy. However, the oxygen levels in the tumor microenvironment were not analyzed in the in vivo experiments, we will systematically supplement the detection of hypoxia-related indicators (such as HIF-1α and tissue partial pressure of oxygen) in future research to further clarify the regulatory effect of the material on the tumor microenvironment and improve the mechanistic explanation.

Developing safe and efficient nanosystems for tumor immunotherapy is critical. In this study, we constructed an OMV-based nanosystem that promotes DCs maturation, as evidenced by CD80 expression, and improves the immunosuppressive tumor microenvironment (TME) through increased CD8^+^ T cell infiltration. The observed immune activation is consistent with TLR pathway involvement, as supported by existing literature. Limitations of this study include unvalidated CD8^+^ T cell cytotoxicity, undetectable levels of key cytokines (IL-12, IFN-γ) and cytotoxic markers (granzyme B, perforin), and insufficient molecular validation of the TLR pathway due to experimental constraints (Ribas and Wolchok, 2018; Qing et al., 2020). Future work will focus on addressing these limitations and optimizing the nanosystem to establish a foundation for clinical translation.

The core advantages of this system lie in the mechanistic linkage between PDT-mediated tumor killing and OMV-based immune regulation, as well as the functional innovation brought by biomineralization modification. In one respect, the CaP shell is not a simple physical coating but realizes the triple functional integration of “toxicity shielding-responsive release-targeted enrichment”, which is different from the single-function optimization of traditional mineralized OMVs and lays a solid carrier foundation for precise therapy. In another respect, its synergistic mechanism is reflected in the key supplementation of OMV-mediated immune activation to PDT. PDT can directly kill OSCC cells, but it has limited ability to eliminate residual lesions and is susceptible to the immunosuppressive effect of TME. In contrast, OMVs can activate DCs to initiate adaptive immunity, promote the infiltration of effector T cells, and reshape the TME, forming a synergistic mode of “PDT direct killing-OMVs clearing residuals and improving TME” to amplify therapeutic effects rather than simply superimposing. Compared with the immune activation mode that relies on the pyroptosis pathway, the pathway centered on DC activation is better suited to the highly heterogeneous TME of OSCC. Moreover, the physicochemical advantages of Ce6, combined with CaP-based targeted modification, are better suited to superficial treatment of OSCC.

## 5 Conclusion

In conclusion, we developed a type of biomineralized OMVs (OCC), which is constructed by coating OMVs with calcium phosphate (CaP) and loading Ce6 onto the shell. OCC shows acidic responsiveness with the shell disintegrating to release OMVs and Ce6 in the acidic environments, thereby achieving synergistic treatment of OSCC via immunotherapy and PDT, and suppressing the tumor growth effectively. Moreover, OCC can reverse the immunosuppressive microenvironment to enhance antitumor immune responses. This spatiotemporal nanostructure maximizes the immunotherapeutic potential of OMVs, overcomes the limitations of traditional monotherapies, and provides an alternative therapeutic approach for the effective treatment of OSCC.

## Abbreviations

**Table:** 

OMVs	Outer membrane vesicles
CaP	calcium phosphate
Ce6	chlorin e6
EPR	enhanced permeability and retention
PDT	photodynamic therapy
OCC	OMVs@CaP-Ce6
OSCC	oral squamous cell carcinoma
ICIs	immune checkpoint inhibitors
CAR-T	chimeric antigen receptor T-cell
TME	tumor microenvironment
PAMPs	pathogen-associated molecular patterns
LPS	lipopolysaccharides
DCs	dendritic cells
TLR4	Toll-like receptor 4
MPS	mononuclear phagocyte system
ROS	reactive oxygen species
SCC7	mouse tongue squamous cell carcinoma
FBS	fetal bovine serum
PBS	phosphate-buffered saline
TEM	transmission electron microscope
PDI	polydispersity index
UV–Vis	ultraviolet-Visible Spectroscopy
SDS-PAGE	sodium dodecyl sulfate-polyacrylamide gel electrophoresis
OC	OMV@CaP
DLS	dynamic light scattering
AAS	atomic absorption spectrometer
CCK8	CCK-8 assay kit
CLSM	confocal laser scanning microscope
PI	propidium iodide
MHC-II	major histocompatibility complex class II molecule
ROI	region of interest
H&E	hematoxylin-eosin

## Availability of data and materials

The datasets used and/or analyzed during the current study are available from the corresponding author upon reasonable request.

## CRediT authorship contribution statement

**Jingyuan Wang:** Writing – original draft, Visualization, Methodology, Investigation, Data curation. **Guanxiong Zhu:** Investigation, Data curation. **Hongru Zhang:** Visualization, Investigation, Data curation. **Liting Zeng:** Supervision, Resources, Funding acquisition. **Da Li:** Investigation. **Xinyi Li:** Visualization, Investigation. **Yang Yu:** Software, Resources. **Lu Liang:** Supervision, Software, Resources, Methodology. **Lingmin Zhang:** Writing – review & editing, Supervision, Software, Resources, Methodology, Conceptualization. **Lina Yu:** Supervision, Software, Resources, Funding acquisition, Conceptualization.

## Ethics approval and consent to participate

Animal experiments were conducted by the Experimental Animal Care Center of Guangdong Laian Technology (Guangzhou) Co., Ltd. (G2025040).

## Funding

This study was supported by the Department of Guangzhou Education Bureau of China (No. 20244312218) and Plan on Enhancing Scientific Research in GMU (2025SRP003).

## Declaration of competing interest

The authors declare that they have no known competing financial interests or personal relationships that could have appeared to influence the work reported in this paper.

## Data Availability

Data will be made available on request.
