# Supplementary material for: Biomineralized outer membrane vesicles for synergistic immuno-photodynamic therapy of oral squamous cell carcinoma
Source: Int J Pharm X. 2026 Apr 7;11:100537. doi: 10.1016/j.ijpx.2026.100537 (PMC13100294; doi:10.1016/j.ijpx.2026.100537)
Supplement: Supplementary file 1 — Supplementary material [file mmc1.docx]

**Supporting information**

**Biomineralized outer membrane vesicles for synergistic immuno-photodynamic therapy of oral squamous cell carcinoma**

Jingyuan Wang ^1,+^,Guanxiong Zhu ^1,+^, Hongru Zhang ^1^, Liting Zeng ^1^, Da Li^1^, Xinyi Li^1^, Yang Yu ^3^, Lu Liang ^1, 2^, Lingmin Zhang ^1, 2,^*, Lina Yu ^1,^*

^1^ Department of Preventive Dentistry, School and Hospital of Stomatology, Guangdong Engineering Research Center of Oral Restoration and Reconstruction & Guangzhou Key Laboratory of Basic and Applied Research of Oral Regenerative Medicine, Guangzhou Medical University, Guangzhou, Guangdong, 510182, People’s Republic of China;

^2^ Guangzhou Municipal and Guangdong Provincial Key Laboratory of Molecular Target & Clinical Pharmacology, the State & NMPA Key Laboratory of Respiratory Disease, School of Pharmaceutical Sciences & the Fifth Affiliated Hospital, Guangzhou Medical University, Guangzhou, 511436, Guangdong, People’s Republic of China;

^3^ Department of Sports and Health, Guangzhou Sport University, Guangzhou, 510500, Guangdong, People’s Republic of China

^+^ There authors contributed to the work equally.

**
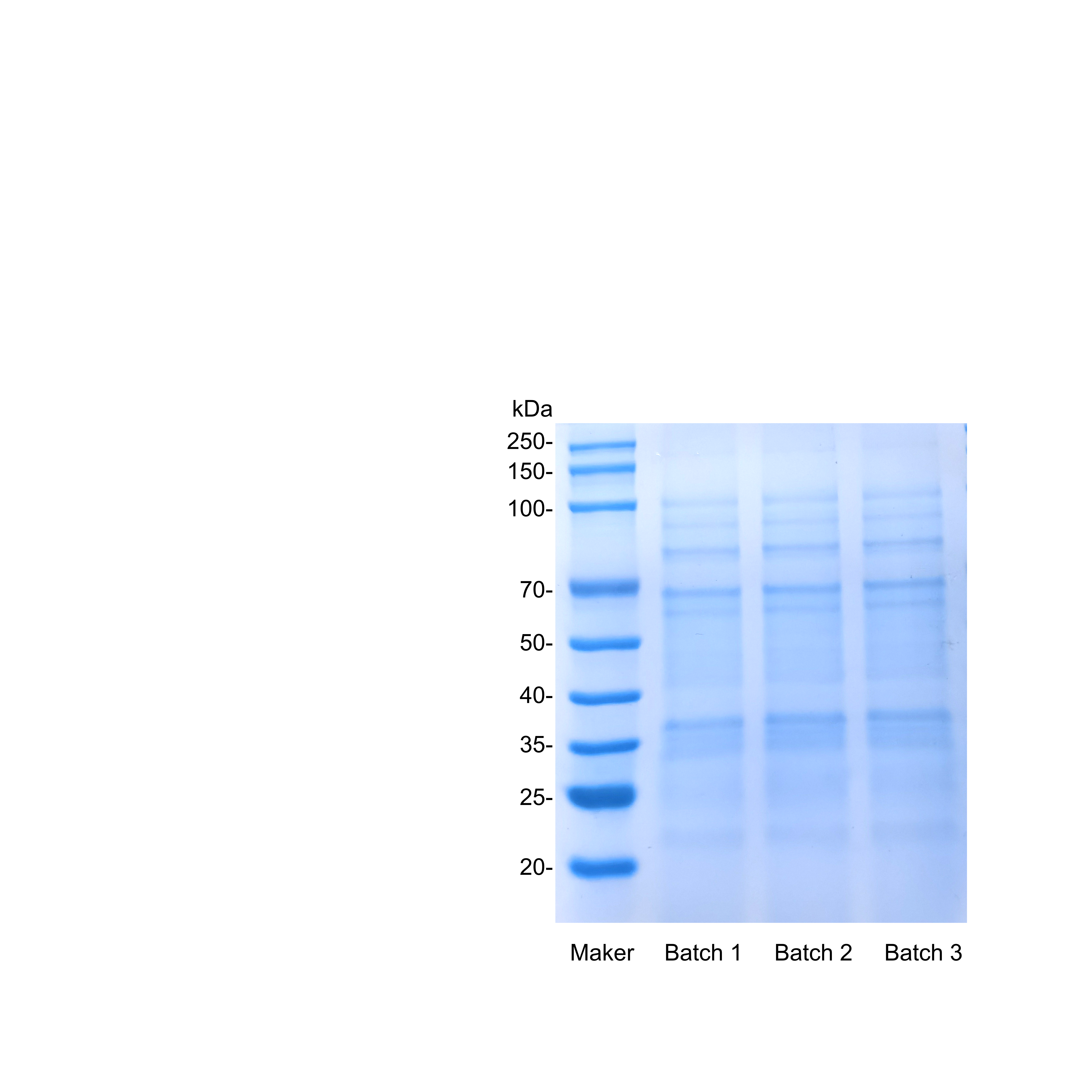
**

**Fig. S1.** SDS-PAGE analysis of protein samples prepared from different batches, followed by Coomassie brilliant blue staining.

**
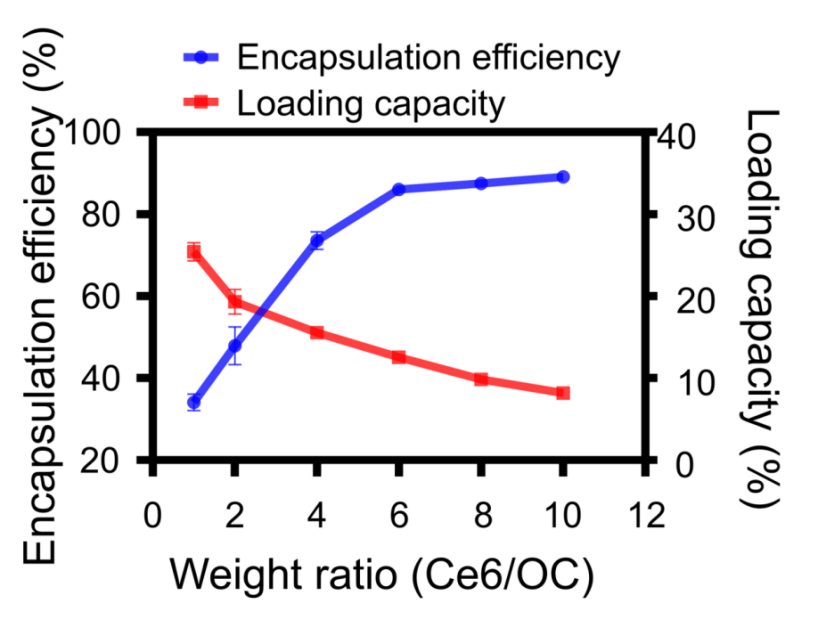
**

**Fig. S2.** The encapsulation efficiency and loading efficiency of Ce6 by OMV@CaP at different Ce6-to-OMV@CaP ratios.

**
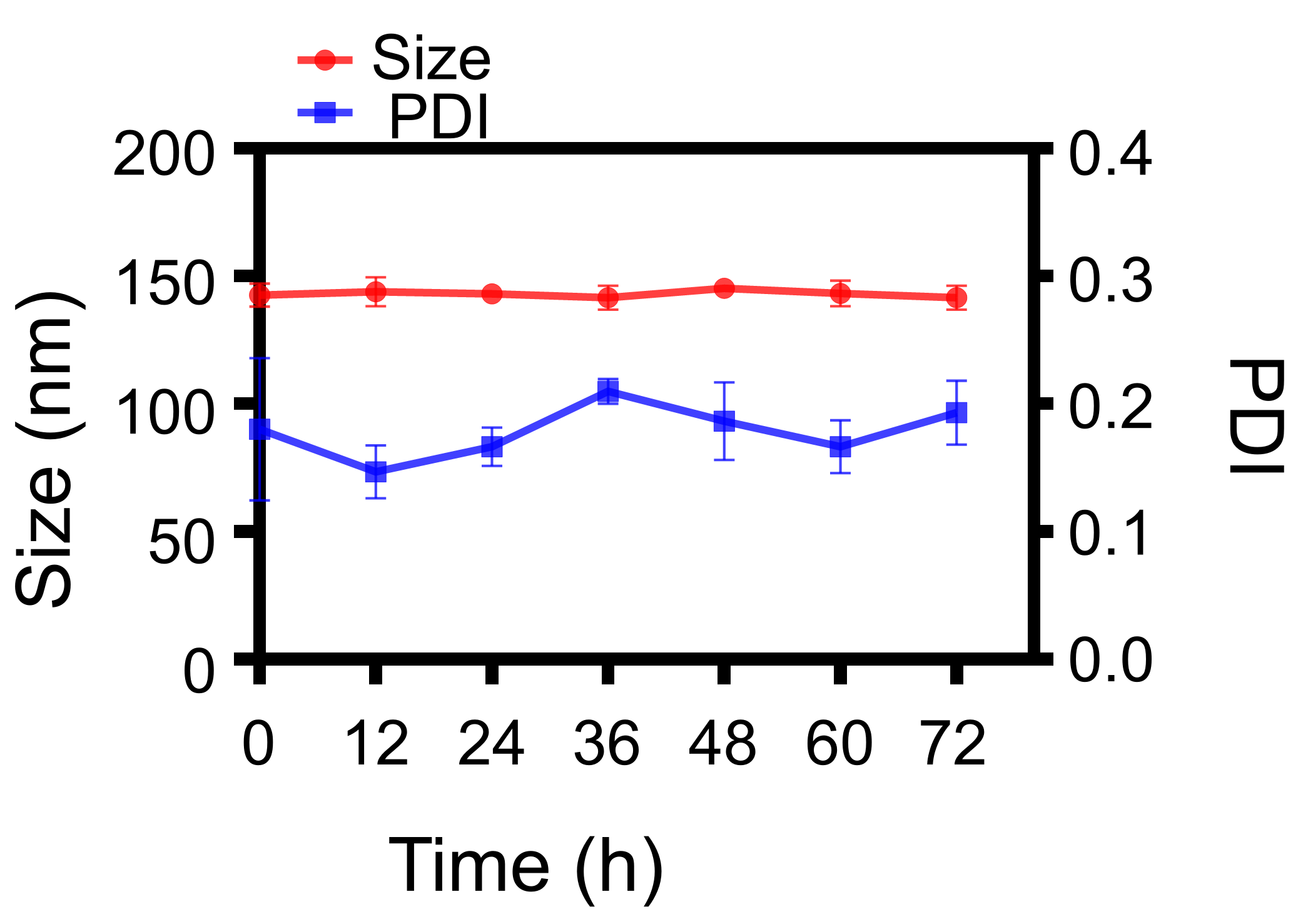
**

**Fig. S3.** The particle size and polydispersity index (PDI) of OCC nanoparticles after incubation for 72 h.

**Table S1**. Fitting results of release kinetics models for pH 7.4 and pH 6.4.

| pH | Model | R² | Release exponent (n) | Release mechanism |
| --- | --- | --- | --- | --- |
| 7.4 | Zero-order | 0.671 | — | — |
|  | Higuchi | 0.864 | — | — |
|  | Korsmeyer-Peppas | 0.980 | 0.629 | Fickian diffusion |
| 6.4 | Zero-order | 0.613 | — | — |
|  | Higuchi | 0.812 | — | — |
|  | Korsmeyer-Peppas | 0.949 | 0.540 | Non-Fickian diffusion |

Notes: The release exponent (n) was calculated from the Korsmeyer-Peppas model using data points with cumulative release ≤ 60%.

**
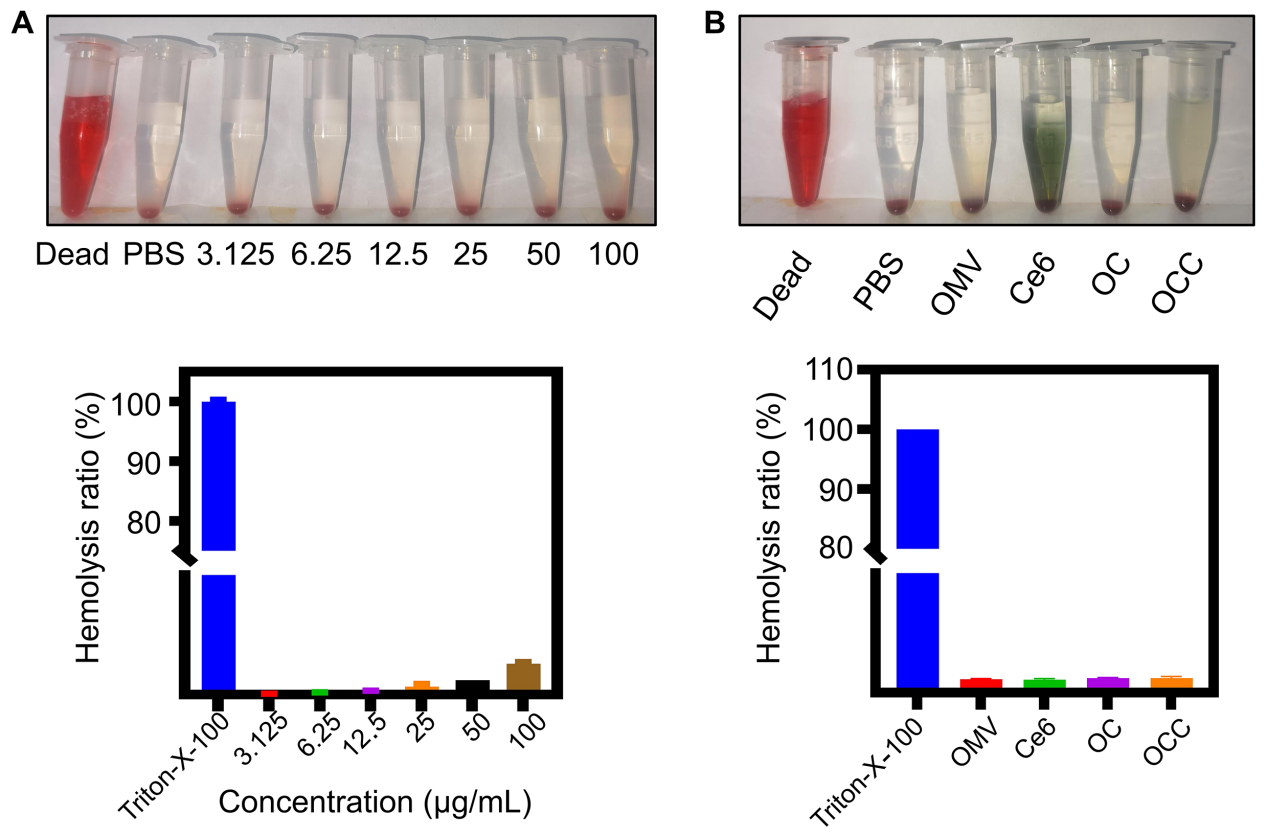
**

**Fig. S4.** In vitro assessment of the biocompatibility of OCC. (A) Quantitative analysis of hemolysis rates was performed using hemolysis assays with varying OCC concentrations (doses of OMV); (B) hemolysis rates were compared across multiple groups.

**
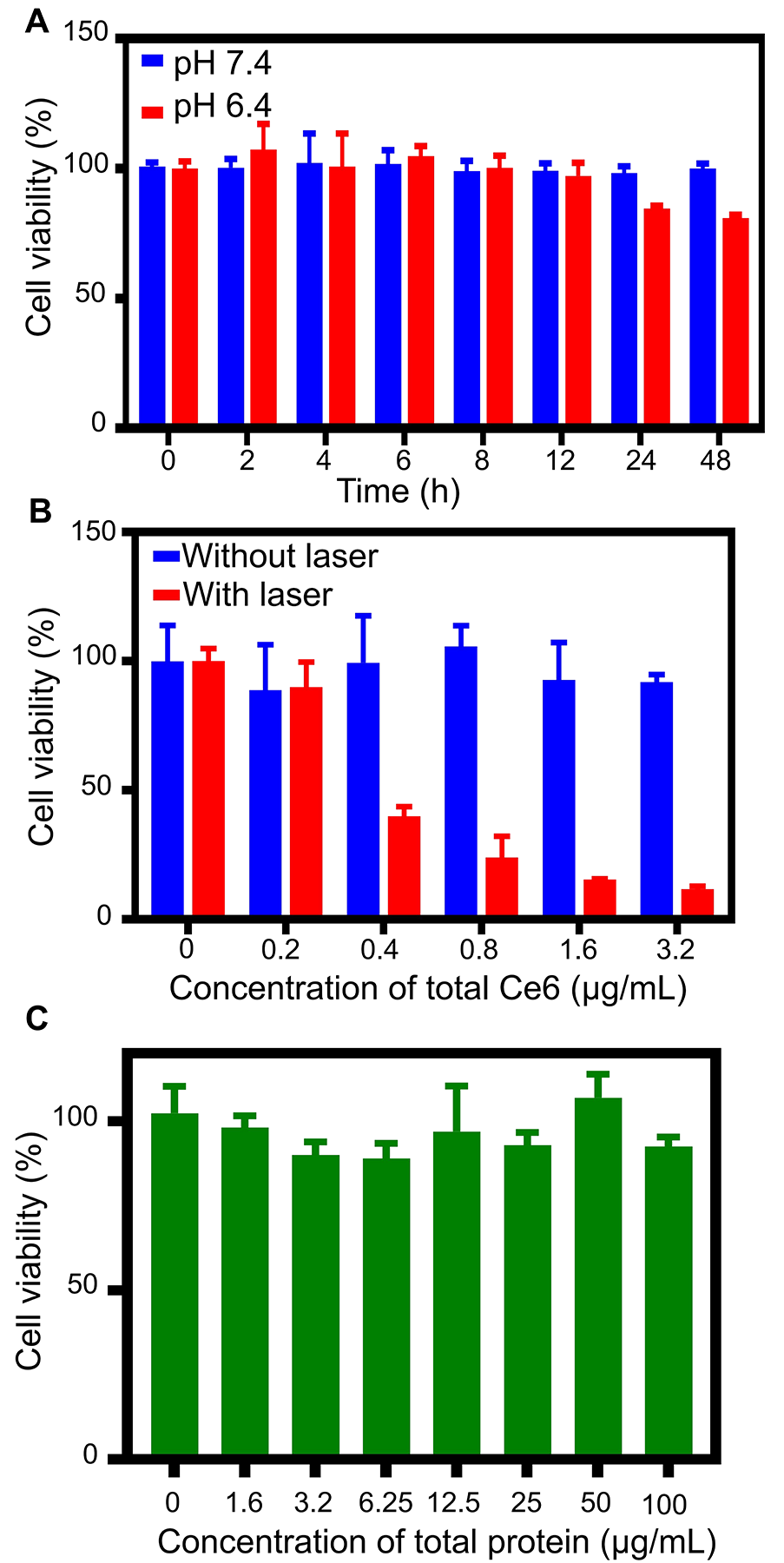
**

**Fig. S5.** In vitro assessments were conducted to evaluate cell cytotoxicity under varying pH levels and OCC concentrations. (A) The viability of SCC7 cells after 48 hours was measured at pH 6.0, and pH 7.4 by the CCK-8 assay. (B) The viability of SCC7 cells was also assessed at different concentrations of OCC, corresponding to doses of Ce6, under two conditions: laser irradiation and no irradiation, using the CCK-8 assay. (C) The survival rate of dendritic cells was analyzed after treatment with varying OCC concentrations (doses of OMV).

**
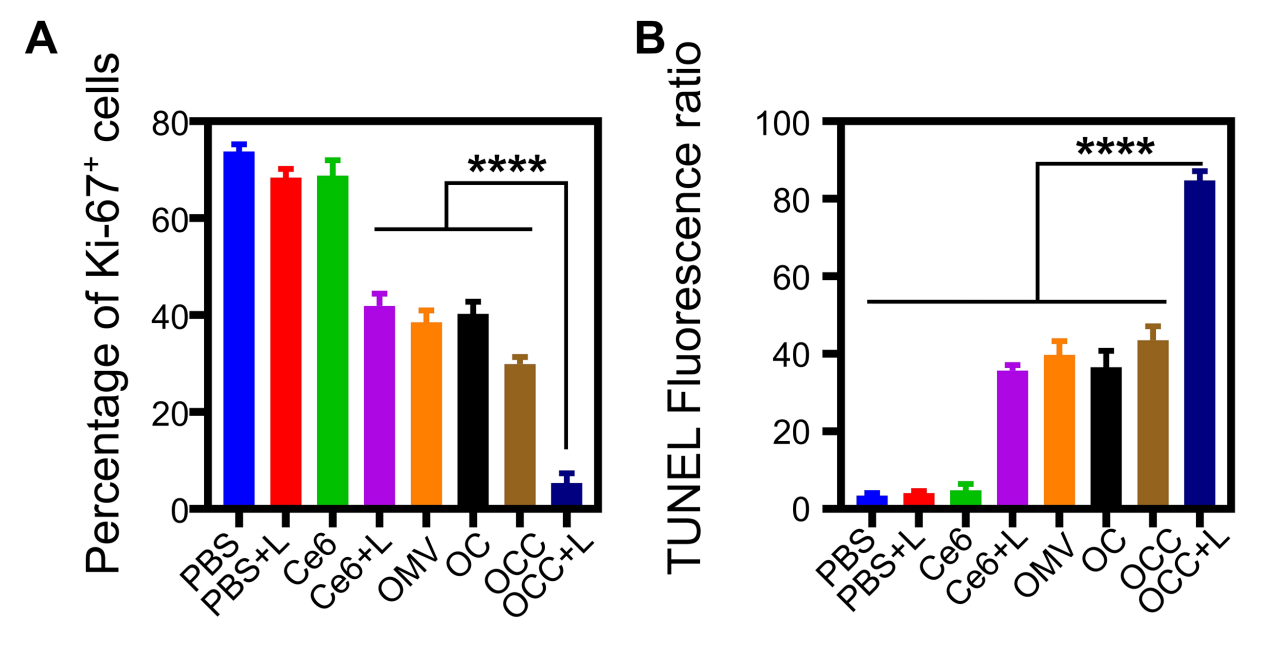
**

**Fig. S6.** Quantitative assessment of Ki-67 (A) and TUNEL (B) in tumors after drug administration was conducted. Statistical significance is indicated as follows: * *P* < 0.05, ** *P* < 0.01, *** *P* < 0.001, **** *P* < 0.0001.


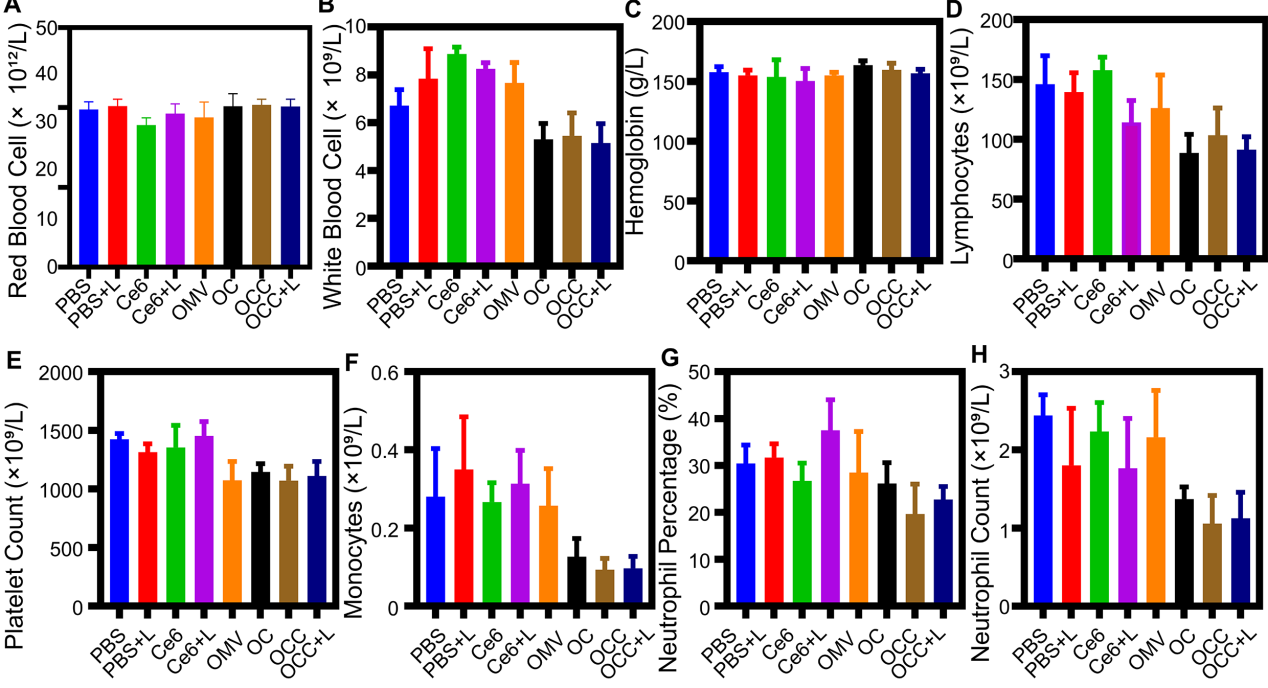


**Fig. S7.** Evaluation of biosafety concerning various nanoparticles requires a systematic analysis of murine blood following different nanoparticle treatment protocols. The parameters assessed include (A) erythrocyte count, (B) leukocyte count, (C) hemoglobin concentration, (D) lymphocyte count, (E) platelet count, (F) monocyte count, and (G) neutrophil count.


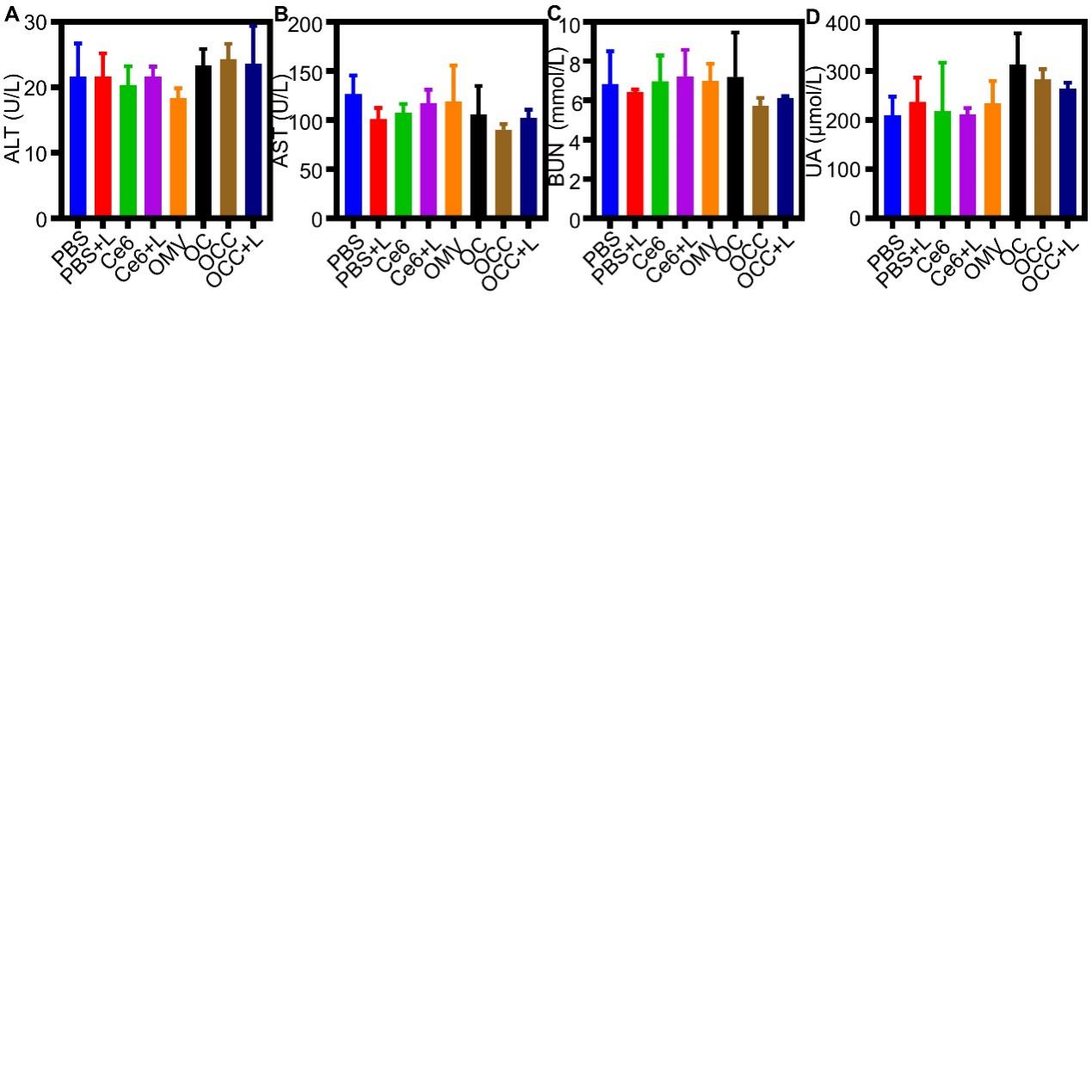


**Fig. S8.** Biosafety assessment of various nanoparticles was conducted by analyzing hepatic and renal functions. Specifically, the levels of hepatic function markers, including (A) alanine aminotransferase and (B) aspartate aminotransferase, as well as renal function indicators, such as (C) blood urea nitrogen and (D) uric acid, were measured to evaluate the potential biological impact of the nanoparticles.

**
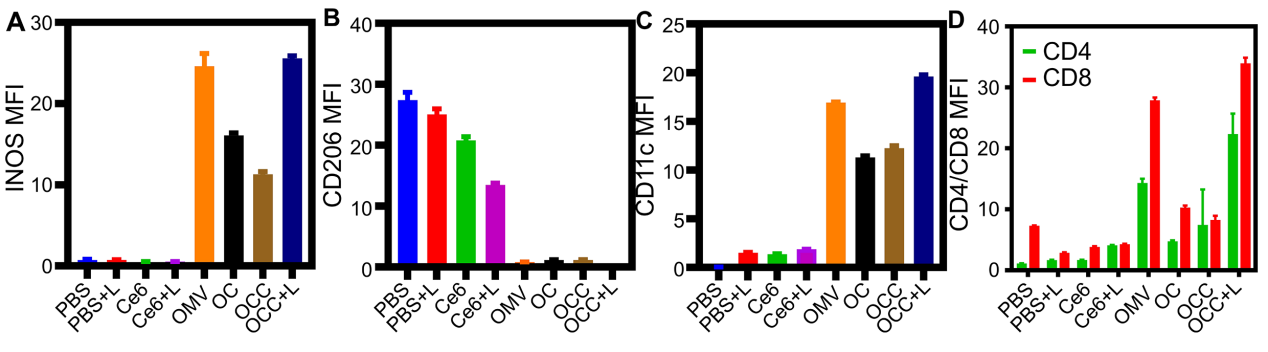
**

**Fig. S9.** The mean fluorescence intensity of (A) iNOS, (B) CD206, (C) CD11c, (D) CD4, and (E) CD8 was quantitatively assessed.
